# PhySpeTree: an automated pipeline for reconstructing phylogenetic species trees

**DOI:** 10.1186/s12862-019-1541-x

**Published:** 2019-12-02

**Authors:** Yang Fang, Chengcheng Liu, Jiangyi Lin, Xufeng Li, Kambiz N. Alavian, Yi Yang, Yulong Niu

**Affiliations:** 1 Key Laboratory of Bio-Resources and Eco-Environment of Ministry of Education, College of Life Sciences, Sichuan University, Chengdu, People’s Republic of China; 2 State Key Laboratory of Oral Diseases & National Clinical Research Center for Oral Diseases &Department of Periodontics, West China Hospital of Stomatology, Sichuan University, Chengdu, China; 3 Wu YuZhang Honors College of Sichuan University, Chengdu, People’s Republic of China; 4 Department of Medicine, Division of Brain Sciences, Imperial College London, London, UK; 5 Department of Internal Medicine, Endocrinology, Yale University, New Haven, USA

**Keywords:** Species tree, Automatic construction, Pipeline

## Abstract

**Background:**

Phylogenetic species trees are widely used in inferring evolutionary relationships. Existing software and algorithms mainly focus on phylogenetic inference. However, less attention has been paid to intermediate steps, such as processing extremely large sequences and preparing configure files to connect multiple software. When the species number is large, the intermediate steps become a bottleneck that may seriously affect the efficiency of tree building.

**Results:**

Here, we present an easy-to-use pipeline named PhySpeTree to facilitate the reconstruction of species trees across bacterial, archaeal, and eukaryotic organisms. Users need only to input the abbreviations of species names; PhySpeTree prepares complex configure files for different software, then automatically downloads genomic data, cleans sequences, and builds trees. PhySpeTree allows users to perform critical steps such as sequence alignment and tree construction by adjusting advanced options. PhySpeTree provides two parallel pipelines based on concatenated highly conserved proteins and small subunit ribosomal RNA sequences, respectively. Accessory modules, such as those for inserting new species, generating visualization configurations, and combining trees, are distributed along with PhySpeTree.

**Conclusions:**

Together with accessory modules, PhySpeTree significantly simplifies tree reconstruction. PhySpeTree is implemented in Python running on modern operating systems (Linux, macOS, and Windows). The source code is freely available with detailed documentation (https://github.com/yangfangs/physpetools).

## Background

The reconstruction of phylogenetic species trees is of central importance in many biological disciplines. For example, the tree of life provides a remarkable view of organizing principles in biology [1, 2]. In addition, many new genomes are being sequenced, and their taxonomic identities can be determined by inserting them into prebuilt species trees [3]. Moreover, combined with species trees, phylogenetic profiling using gain and loss patterns of homologs achieves high performance in predicting protein linkages [4–8].

Toolkits and pipelines have been developed for phylogenetic reconstruction (Table 1). Toolkits such as BuddySuite [9], ETE3 [10], and MEGA [11] are widely used for phylogenetic inference and tree manipulation. BuddySuite and ETE3 provide rich interfaces that allow researchers to carry out secondary development. BuddySuite includes a pipeline with which to reconstruct gene or species trees, but third-party software needs to be specified and manually installed in the local running environment, which may be inconvenient for users on different platforms. MEGA is a standalone and cross-platform program, and it also provides a user-friendly graphical interface. BIR [12], Agalma [13], PhyloPlAn [14], and AMPHORA [12] are designed for phylogenomic analysis. BIR is particularly useful for preparing gene sequences for phylogenomic inference. Agalma has a command-line interface for phylogenomic analyses based on genomic and transcriptome data. PhyloPlAn and AMPHORA (AMPHORA2 [14]) are effective pipelines for large-scale phylogenetic inference based on thoroughly tested marker genes, and other operations such as taxonomic curation, estimation, and insertion are also available. The marker genes, however, are conserved only between microbial genomes, so PhyloPlAn and AMPHORA are limited to reconstructing bacterial and archaeal species trees.

**Table 1:**
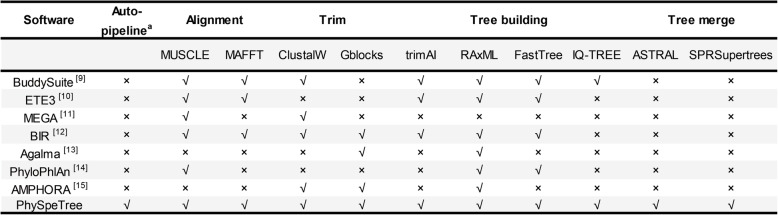
Comparison of phylogenetic tree construction software

^a^ The automation refers to sequences download, preprocess, and preparation of configure files. Critical steps such as sequence alignment and tree construction can be manually adjusted with advanced options in PhySpeTree

Although the software mentioned above are powerful in inferring phylogenies, most require users to manually download genomic data, clean and align sequences, or prepare complex configure files. These laborious and time-consuming steps may impede tree reconstruction, especially when the number of species becomes large. Hence, there is a clear need for a flexible and efficient pipeline that can reduce the time required for species tree building processes.

Here, we present an easy-to-use Python package named PhySpeTree, which provides an automated solution for the entire process of species tree reconstruction, from data collection to tree building. PhySpeTree has two parallel pipelines based on either the most commonly adopted small subunit ribosomal RNA (SSU rRNA) [15] or concatenated highly conserved proteins (HCPs) [16]. The distinguishing feature of PhySpeTree is its automated design. Users need only to input the abbreviations of species names, and then PhySpeTree can automatically download and analyze sequences. Some critical steps, such as multiple sequence alignment and tree construction, can be manually adjusted. Moreover, PhySpeTree contains modules to facilitate downstream analysis. For example, users can apply the “autobuild” module to extend prebuilt trees by inserting new organisms. The “iview” and “combine” modules are designed for tree visualization in iTOL [17] and consensus tree construction [18], respectively. Together with accessory modules, PhySpeTree significantly simplifies tree reconstruction.

### Implementation

**Fig. 1:**
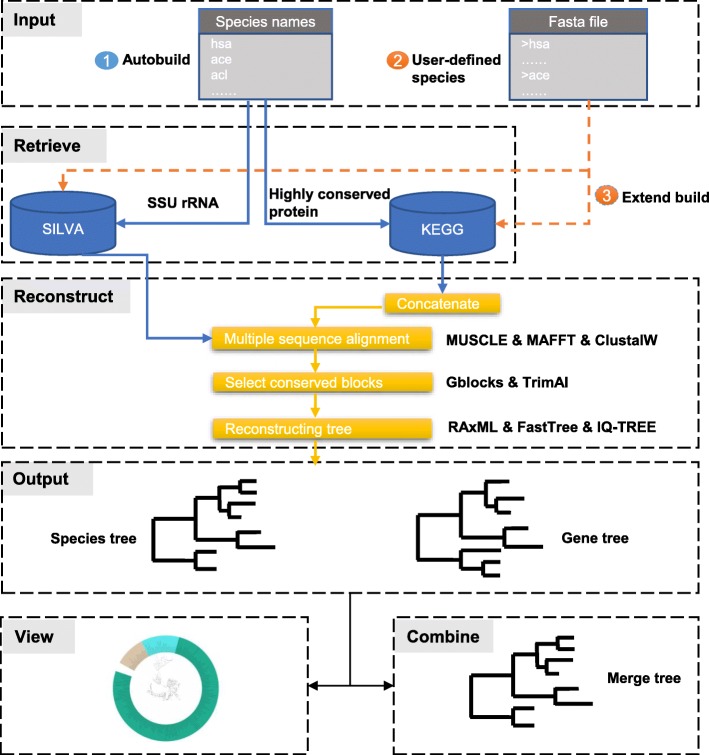
The workflow of PhySpeTree. PhySpeTree retrieves and downloads corresponding sequences following with multiple sequence alignments, conserved blocks selection, and tree reconstruction. PhySpeTree also allows users to insert their own sequences of HCP or SSU rRNA. Output trees are in the “newick” format files. ①Automatic tree reconstruction ②Processing user-defined fasta files for unannotated organisms ③Reconstructing species trees with unannotated organisms

PhySpeTree is implemented in Python and distributed as an independent package. PhySpeTree integrates multiple tools and provides an automated solution for reconstructing species trees (Table 1). The workflow of PhySpeTree is shown in Fig. 1. First, users input the abbreviations of species names (Additional file 1: Figure S1 and Additional file 2: Table S2) and choose the sequence type (SSU rRNA or HCP) to build species trees. If the HCP option is selected, PhySpeTree retrieves and concatenates HCP sequences from the Kyoto Encyclopedia of Genes and Genomes (KEGG) database [19]. Otherwise, PhySpeTree uses SSU rRNA sequences from the SILVA database [20]. For unannotated organisms, users can prepare FASTA format files containing either HCP or SSU rRNA sequences and then insert them into prebuilt databases. Second, multiple sequence alignment is conducted by MUSCLE [21], MAFFT [22], or ClustalW [23], and conserved blocks are selected by Gblocks [24] or trimAI [25]. Finally, PhySpeTree reconstructs species trees by RAxML [18], IQ-TREE [26], or FastTree [27]. In addition, PhySpeTree provides flexible modules to facilitate downstream analysis, such as generating visualization files for iTOL [17] and tree combination (Fig. 1).

### SSU rRNA option

For bacterial and archaeal organisms, SSU rRNA sequences are widely used to build species trees [2]. We prebuilt a dataset according to the latest version of the SILVA database (Release 132, Dec. 13, 2017) [20]. The dataset contains truncated SSU rRNA sequences from 140,662 species, and nucleotides that are not aligned are removed (Additional file 1: Figure S1A and Additional file 2: Table S1). When the SSU rRNA option is selected, PhySpeTree automatically fetches related sequences.

### HCP option

It has been reported that HCP-based species trees have a higher resolution than the ones built based on a single gene [15]. Hence, PhySpeTree also provides the HCP option. First, we chose 31 single-copy HCPs without horizontal transfer from Ciccarelli et al [16]. Then, we manually mapped them to KEGG orthologues (Release 90.1, May 1, 2019) [19] (Additional file 2: Table S3). When users choose the HCP option, PhySpeTree directly retrieves HCP sequences from the KEGG database. The HCP option currently supports 5943 organisms (Additional file 1: Figure S1B and Additional file 2: Table S2).

### Sequence alignment and tree reconstruction

PhySpeTree integrates various tools for multiple sequence alignment and tree reconstruction. For sequence alignment, MUSCLE [21], MAFFT [22], and Clustal [22] are provided. To infer accurate phylogenies, the maximum likelihood-based method RAxML is set as the default option [18]. In addition, IQ-TREE [26] and FastTree [27] are alternatives to accelerate tree reconstruction. Advanced parameters of integrated tools can be specifically set and passed in PhySpeTree, allowing users to manipulate critical steps in sequence alignment and tree reconstruction.

## Result

### Modules of PhySpeTree

PhySpeTree contains five modules. The main module “**autobuild**” is developed to automatically build species trees. With this module, users do not need to prepare sequences in advance. Instead, the abbreviations of species names are the only required inputs. The intermediate steps, e.g., sequence download, cleaning, alignment, and tree reconstruction, are automatically handled by PhySpeTree. The following command line shows an example:

#### $ PhySpeTree autobuild -i species_names.txt --hcp

where “species_names.txt” is the file of abbreviated organism names; for example, “hsa” represents *Homo sapiens* (Additional file 2: Table S2). “--hcp” indicates that the HCP option is selected.

Moreover, the “**autobuild**” module can be used to extend prebuilt trees by inserting new organisms whose genome annotations may be incomplete. For the new organisms, the SSU rRNA may come from experiments, while orthologous databases such as eggNOG [28] and OMA [29] are good resources for searching for corresponding HCP sequences. FetchMG [30] is also available to identify HCP sequences in reference genomes and metagenomes. The following commands illustrate how to insert a new organism into trees:

#### $ PhySpeTree autobuild -i species_names.txt -e new_hcp.fasta --ehcp

where “new_hcp.fasta” is the HCP sequence of the new organism. The file should be prepared by users. “--ehcp” indicates that the tree is extended according to HCP sequences.

Instead of using default settings, in the “**autobuild**” model, users can adjust advanced options to control critical steps, such as sequence alignment, conserved block selection, and tree building. The following command shows how to set advanced options of RAxML:

#### $ PhySpeTree autobuild -i species_names.txt --srna --raxml --raxml_p ‘-f a -m GTRGAMMA -p 12345 -× 12,345 -# 100 -n T1’

where “--raxml_p” indicates advanced options passed to RAxML.

The module “**build**” is developed for advanced users to directly reconstruct trees from protein or gene sequences. It is practically useful to reconstruct trees by user-defined sequences other than SSU rRNA or HCP sequences. This function may overlap with ETE3 and MEGA and is executed as:

#### $ PhySpeTree build -i defined_seq.fasta --single

where “defined_seq.fasta” is a FASTA file containing user-defined sequences. “--single” indicates a single sequence for each organism.

The “**iview**” module is designed to facilitate tree visualization. It provides a convenient interface used to generate configure files for iTOL, which is a powerful online tool for tree display, annotation, and manipulation [17]. The taxonomy of species is directly retrieved from the KEGG database. The following command annotates input species at the phylum level:

#### $ PhySpeTree iview -i species_names.txt --range -a phylum

where “species_names.txt” is the same file as in the “autobuild” module.

To reconstruct a consensus tree, PhySpeTree uses the “**combine**” module to merge multiple trees. This module is useful for comparing and selecting conserved branches from trees generated by different sequences or tree building methods. It is implemented as follows:

#### $ PhySpeTree combine -i combine.tree

where “combine.tree” is a file containing multiple trees.

The module “**check**” is designed to check whether input species are supported in PhySpeTree. It can also be used to check sequence information that is needed to extend the current tree. The following command will return species that are not supported by the HCP option:

#### $ PhySpeTree check -i species_names.txt –hcp

##### Installation

The PhySpeTree pipeline is implemented in Python and has been tested on Linux systems such as Fedora, Ubuntu, and CentOS. We also released a Docker image to support Windows and macOS. The latest version can be installed as follows:

#### $ pip install PhySpeTree

Alternatively, PhySpeTree can be directly installed from the GitHub repository. Code is available at https://github.com/yangfangs/physpetools/releases, and PhySpeTree is installed by a local command as follows (executed in the PhySpeTree directory):

#### $ python setup.py install

##### Usage and tutorial

To facilitate the use of PhySpeTree, we distribute a detailed tutorial (https://yangfangs.github.io/physpetools/) (Additional file 3). The tutorial provides step-by-step examples to show how to use the modules mentioned above.

### Benchmark test of the efficiency and consistency of PhySpeTree

To test the efficiency of PhySpeTree, we simulated five data sets with different numbers of species (50, 100, 300, 600, and 1000) by randomly selecting taxa from our prebuilt HCP and SSU rRNA databases. Each data set was independently generated three times, and the mean and standard deviation of the run time were recorded. One of the great advantages of using PhySpeTree is that it provides automated sequence preprocessing (e.g., querying databases, downloading sequences, and formatting). The time required for this process showed linear growth with an increase in the number of species (Table 2). It took approximately 3 s to preprocess one species, and most of the time was spent on querying remote prebuilt databases. The prebuilt databases can be downloaded and easily deployed, so we provided a special option, “-db”, to further improve efficiency by manipulating sequences on local computers. Another advanced feature of PhySpeTree is the fully optimized configuration of software streams. We then compared the run time of tree building in PhySpeTree with that of a pipeline in BuddySuit [9]. The same third-party software and parameters were used. In PhySpeTree, the SSU rRNA option was slightly better than the HCP option, mainly because more sequences were processed with the HCP option. The run time of BuddySuit was comparable to that of PhySpeTree when building small tress (fewer than 300 species), whereas PhySpeTree outperformed BuddySuit when the number of species increased. For example, in building a tree with 1000 species, the optimized configuration of PhySpeTree resulted in more than a 5X speed gain (Table 2). Overall, our benchmark tests showed that PhySpeTree is a highly efficient pipeline in the reconstruction of large-scale trees.

**Table 2:**
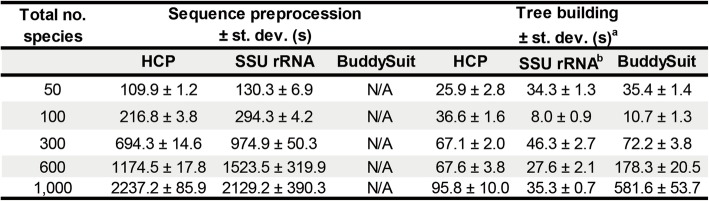
Run time test of PhySpe Tree

^a^ The tree reconstruction pipeline was conducted by MAFFT (alignment), Gblocks (trim), and FastTree (tree building). Benchmark test was conducted with i7-4790 3.6GHz CPU (parallel on 6 threads) with 16GB memory on Fedora operating system. ^b^ "--auto" option was turn on in MAFFT. The alignment strategy was automatically chose according to the number and length of sequences

There is a lack of ground truth for evaluating the topological accuracy of phylogenies across a wide range of species. As a surrogate, we quantitatively assessed the consistency of species trees from PhySpeTree with respect to the updated tree of life [31]. Because most organisms in the tree of life were uncultured or newly identified, we manually checked and filtered species names from our prebuilt databases. Finally, the SSU rRNA and HCP options matched 154 and 122 species, respectively (Additional file 2: Table S4 and Table S5). We then randomly selected 20, 50, and 100 species and used PhySpeTree to reconstruct species trees with both the SSU rRNA and HCP options. Normalized Robinson-Foulds (nRF) distances, ranging from 0 (identical) to 1 (most unlikely), were calculated to measure topological similarity (Table 3) [10, 32]. Unsurprisingly, SSU rRNA trees archived near perfect consistency (mean nRF distance < 0.13) with the tree of life, as almost identical SSU rRNA sequences were used. For up to 100 species, we found that the HCP option of PhySpeTree was also feasible (mean nRF distance: 0.18 ~ 0.32). Notably, increasing the number of species did not significantly reduce the accuracy of tree reconstruction. The topological dissimilarity between HCP trees and the tree of life was mainly due to the number and type of conserved proteins used to reconstruct the trees.

**Table 3:**
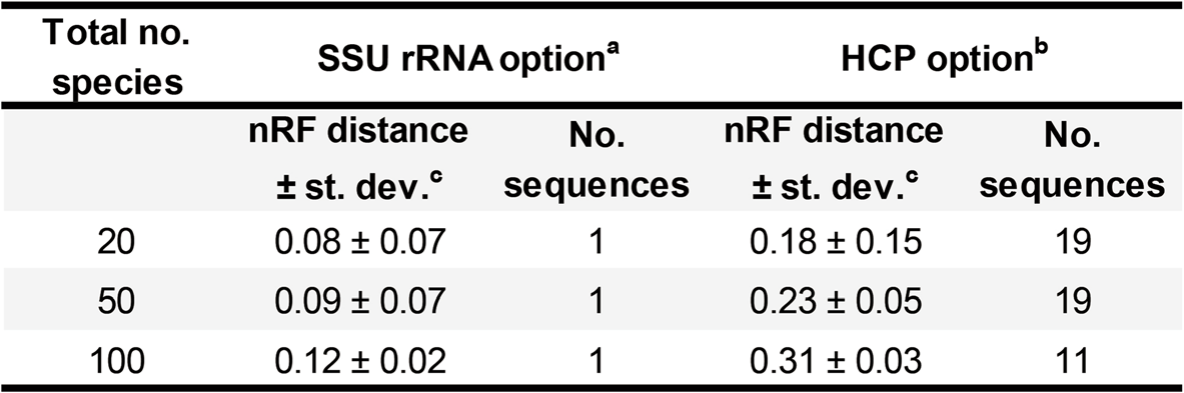
Consistency test of PhySpe Tree comparied with the updated tree of life [33]

^a^ SSU rRNA sequences were retrieved by PhySpeTree, aligned by SINA, and tree reconsturction by RAxML (GTRCAT model). ^b^ HCP sequences were retrieved by PhySpTree, aligned by MUSCLE, and tree reconstruction by RAxML (PROTGAMMAJTTX model). ^c^ Normalized Robinson-Foulds (nRF) distance was calculated by ETE3 [10]

### Case study: the evolutionary position of the archaeal phylum *Lokiarchaeota*

A recent study reported a novel archaeal phylum, *Lokiarchaeota*. Genomes in this phylum encode various eukaryotic signature proteins. Further phylogenetic analysis revealed a close relationship between *Lokiarchaeota* and Eukarya [3]. However, debates about the *Lokiarchaeota*-Eukarya affiliation arose mainly due to the number of species and HCPs used in tree reconstruction [3, 33]. PhySpeTree can be conveniently applied to investigate the evolutionary position of newly identified organisms. Thus, here, we provide an example to show how to insert *Lokiarchaeum sp.* GC14_75 (*loki*) into prebuilt species trees.

**Fig. 2:**
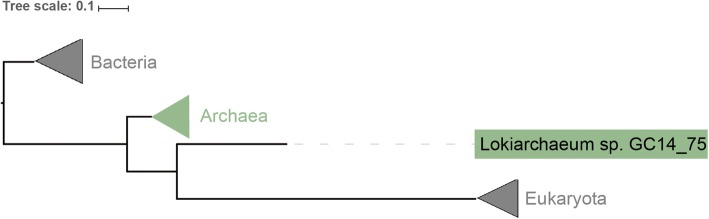
Extended tree of life with *Lokiarchaeota*. The *Lokiarchaeum sp.* GC14_75 was inserted into the tree of life. The bacterial, eukaryotic, and archaeal branches were clasped

At first, we randomly chose 1246 species, including 440 eukaryotic, 544 bacterial, and 280 archaea species, from the prebuilt HCP database, then used PhySpeTree to reconstruct a species tree with the HCP option (Additional file 2: Table S6). Next we prepared 25 HCPs of *loki* (ribosomal protein L1/L3/L5/L11/L13/L14/L22/ S2/S3/S4/S5/S7/S8/S9/S11/S13/S15/S17, phenylalanine−/seryl−/leucyl−/arginyl-tRNA synthetase, metal-dependent proteases with chaperone activity, predicted GTPase probable translation factor, and preprotein translocase subunit SecY) and used the “autobuild” module (with the “-e” option) to expand the tree of life with *loki* (Fig. 2 and Additional file 4). In accordance with the previous species trees [34, 35] reconstructed based on 55 concatenated ribosomal proteins, our results indicated phylogenetic affiliation between *loki* and eukaryotes. Although our results did not support that *loki* and other archaeal lineages were monophyletic [36], various tree topologies can be easily explored by PhySpeTree with different HCPs.

## Conclusions

We developed an automated pipeline named PhySpeTree to reconstruct species trees across bacteria, archaea, and eukaryotes. The PhySpeTree pipeline contains as many options as other tree-building tools (detailed comparison in Table 1). However, another feature sets PhySpeTree apart: it automates intermediate processes, including retrieving sequences from public databases, preparing complex configure files to run different software, aligning sequences, and building trees. The inputs of PhySpeTree are simple that users need only to prepare the abbreviations of species names. For unannotated organisms, users can apply the “check” and “autobuild” modules in PhySpeTree to prepare sequence files. Because PhySpeTree is frequently synchronized with the most recent public databases, the number of unannotated organisms is expected to be small.

PhySpeTree provides both the traditional SSU rRNA option and the HCP option to reconstruct species trees. Benefiting from comprehensive rRNA databases (e.g., SILVA and RDP) [20, 37] and high-throughput rRNA amplicon sequencing [38], SSU rRNA has been widely used as a phylogenetic marker for taxonomic identification [31, 39]. However, inferring taxonomies based on a single marker gene is challenging, given that chimeric sequences arising from PCR and sequencing errors can corrupt tree topologies [40] as well as the limited resolution of SSU rRNA in closely related species [41]. Compared with trees obtained from a single marker gene, those reconstructed by the concatenation of highly conserved single-copy proteins show a higher resolution [16, 31, 42]. For example, to explore the phylogenetic history of organisms, a species tree across all three domains of life was generated based on HCPs [16]. The same set of HCPs was applied for the species assignment of prokaryotic genomes [43] and to establish metagenomic operational taxonomic units [44] and is applied in the HCP option in PhySpeTree. Recently, several revised species trees have been inferred by the concatenation of 16 ribosomal proteins [31] or 120 bacterial proteins [45, 46] to explore the tree of life. Although HCPs are extensively used, when applying the HCP option of PhySpeTree, users should be aware of the limitations of HCPs, such as recombination [42] and potential lateral gene transfer [47].

PhySpeTree was developed in Python and is executed as command lines, so it is easy for advanced developers to expand its modules or integrate PhySpeTree with other phylogenetic tools. For example, PUmPER [48] updates existing trees with new gene sequences. PhySpeTree may work as a complementary tool in terms of building the initial tree and automatically preparing updated sequences from public databases. On the other hand, users of PhySpeTree are reminded that phylogenetic discordance mainly caused by different evolutionary processes affects species tree accuracy [49]. Coalescent-based methods are broadly used to address incongruence [50]. ASTRAL [51] and NJst [52] are efficient tools for handling incomplete lineage sorting. They are also robust to branch length errors, which may result from rate heterogeneity. PhyloNet [53, 54], iGTP [55], Guenomu [56], and SPRSupertrees [57] consider gene flow, gene duplication and loss, or horizontal gene transfer when inferring species trees. The coalescent-based methods mentioned above take gene trees as inputs, which can be conveniently estimated by PhySpeTree (“build” module) or any other tool listed in Table 1. Moreover, species trees inferred from PhySpeTree can benefit from other types of evidence; for example, fossils and ancient DNA can be incorporated into node-based and tip-based calibration [58, 59].

## Availability and requirements

**Project name:** PhySpeTree

**Project home page:** https://yangfangs.github.io/physpetools/

**Operating systems:** Linux (Docker image for Windows and macOS)

**Programming language:** Python 2.7+ and python 3+

**License:** GNU General Public License v3.0

**Other requirements:** None

## Supplementary information

**Additional file 1: Figure S1.** The taxonomic distribution of species supported by the HCP (A) and SSU rRNA options (B).

**Additional file 2: Table S1.** The list of 140,662 species supported in the SSU rRNA option. **Table S2.** The list of 5943 species supported in the HCP option. **Table S3.** The list of 31 highly conserved proteins and corresponding KEGG IDs. **Table S4** and **Table S5.** The lists of SSU rRNA and HCP matched species between prebuilt databases of PhySpeTree and the updated tree of life, respectively. **Table S6.** Species used to reconstruct the tree of life in Fig. 2.

**Additional file 3.** The step by step usage and tutorial for PhySpeTree.

**Additional file 4. **Data used to extend tree-of-life with *Lokiarchaeum sp.* GC14_75. “FastTree.tree” is the output tree file. “tree_of_life_species_names_abb.txt” contains the species abbreviated names to use reconstruct tree-of-life. “highly_conserved_protein_loki” contains *Lokiarchaeum sp.* GC14_75 HCP sequences. “parameter.txt” contains parameter commands.

## Data Availability

The datasets supporting the conclusions of this article are included within the article and its additional files.

## Abbreviations

HCP: Highly Conserved Protein
RNA; KEGG: Kyoto Encyclopedia of Genes and Genomes
SSU rRNA: Small subunit ribosomal
