# Supplementary figures and images for: PhySpeTree: an automated pipeline for reconstructing phylogenetic species trees
Source: BMC Evol Biol. 2019 Dec 2;19:219. doi: 10.1186/s12862-019-1541-x (PMC6889546; doi:10.1186/s12862-019-1541-x)

**A**

Species supported in the SSU-rRNA option

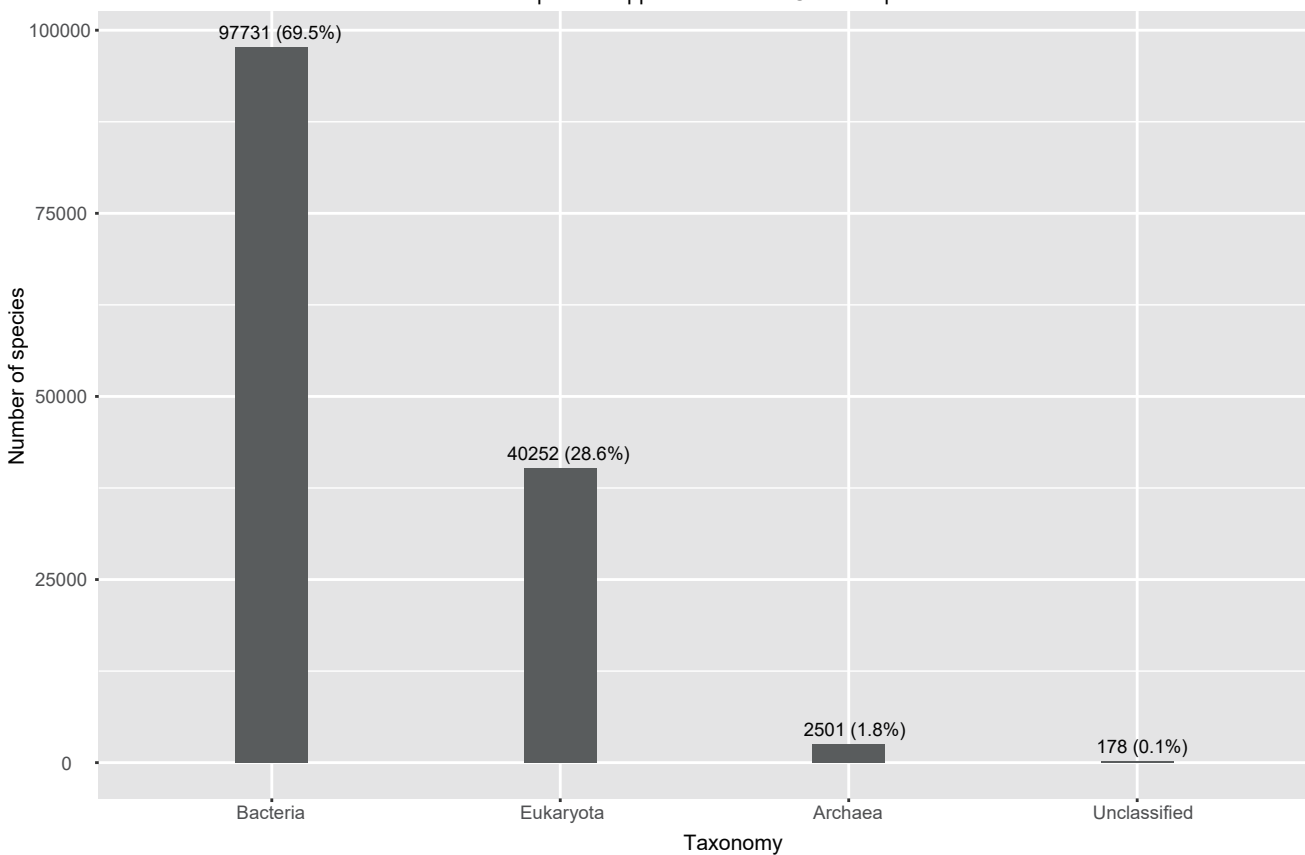**B**

Species supported in the HCP option

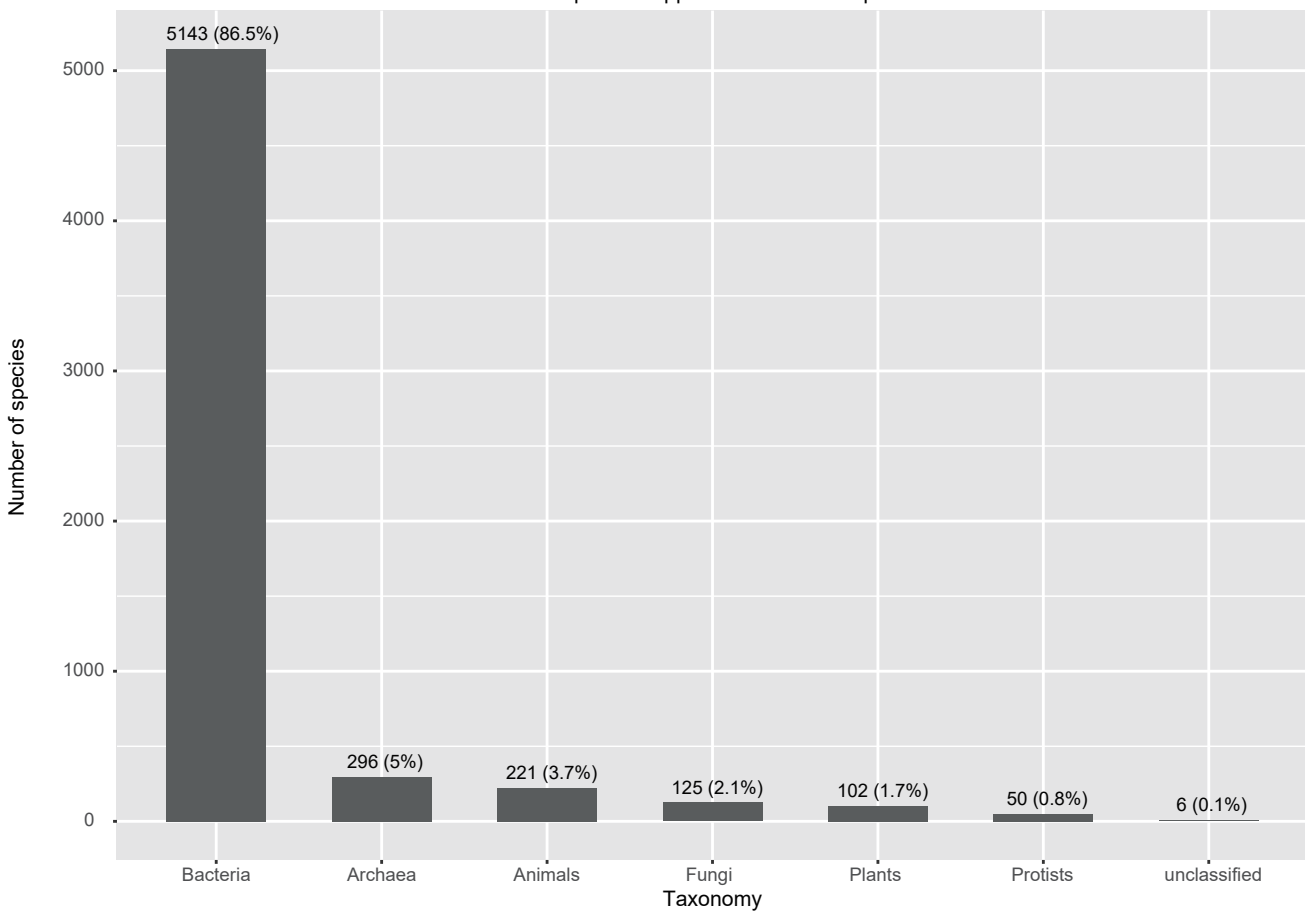

Supplement: Supplementary file 2 — Additional file 2: Table S1. The list of 140,662 species supported in the SSU rRNA option. Table S2. The list of 5943 species supported in the HCP option. Table S3. The list of 31 highly conserved proteins and corresponding KEGG IDs. Table S4 and Table S5. The lists of SSU rRNA and HCP matched species between prebuilt databases of PhySpeTree and the updated tree of life, respectively. Table S6. Species used to reconstruct the tree of life in Fig. 2. [file 12862_2019_1541_MOESM2_ESM.pdf]
