# Supplementary material for: PhySpeTree: an automated pipeline for reconstructing phylogenetic species trees
Source: BMC Evol Biol. 2019 Dec 2;19:219. doi: 10.1186/s12862-019-1541-x (PMC6889546; doi:10.1186/s12862-019-1541-x)
Supplement: Supplementary file 3 — Additional file 3. The step by step usage and tutorial for PhySpeTree. [file 12862_2019_1541_MOESM3_ESM.pdf]

Manual

Contents

Usage and Examples 2
- autobuild 2
  - options 2
  - Example 3
  - Advanced options 8
- build 10
  - build options 11
  - Example 11
- combine 16
  - combine options 16
  - Example 17
  - Using `--astral` option 18
- Using `--supertree` option 19
- iview 19
  - iview options 20
  - Example 20
- check 24
  - check options 24
  - Example 24

PhySpeTree Tutorial 31
- Automatically reconstruct the tree-of-life 31
  - Reconstruct the tree-of-life by the HCP method 31
  - Reconstruct the tree-of-life by the SSU rRNA method 39
- Automatically reconstruct *Plantae* species tree 50
- Extend species tree with new organisms 58
  - Extend species tree by the SSU rRNA method 58
  - Extend species tree by the HCP method 64

Run PhySpeTree in other operating systems ... 68
1. Install the Docker in your operating system ... 68
2. Pull the PhySpeTree image ... 68
3. check PhySpeTree image ... 69
4. run PhySpeTree image ... 69
5. Run PhySpeTree in Docker ... 69

Usage and Examples

autobuild

The input of `autobuild` module is a TXT file containing KEGG abbreviated species names, for example organism_example_list.

```
$ PhySpeTree -i autobuild organism_example_list.txt [options]*
```

options

option	Description
-h	Print help message and exits.
-i	Input a TXT file containing abbreviated species names.
-o	A directory to store outputs. The default is "Outdata".
-t	Number of processing threads (CPUs). The default is 1.
-e	FASTA format files to extend the tree with the --ehcp or --esrna option.

option	Description
-db	The absolute path for local database.
--hcp	HCP (highly conserved protein) method (default).
--ehcp	HCP method with extended HCP sequences.
--srna	SSU method.
--esrna	SSU rRNA method with extended SSU rRNA sequences.

Example

Download the example input file:

```
$ wget "https://yangfangs.github.io/physpetools/example/organism_example_list.txt"



--2016-10-29 19:41:53--  https://yangfangs.github.io/physpetools/example/organism_example_list.txt

Resolving yangfangs.github.io (yangfangs.github.io)... 151.101.24.133

Connecting to yangfangs.github.io (yangfangs.github.io)|151.101.24.133|:443... connected.

HTTP request sent, awaiting response... 200 OK

Length: 39 [text/plain]

Saving to: ‘organism_example_list.txt’



organism_example_list.txt    100%[=============================================>]     39  --.-KB/s    in 0s



2016-10-29 19:41:54 (19.0 MB/s) - ‘organism_example_list.txt’ saved [39/39]
```

```
$ cat organism_example_list.txt
aca
ace
acl
acn
aco
acp
adg
adk
aeh
aeq
```

Automatically reconstruct species trees by HCP

```
$ PhySpeTree autobuild -i organism_example_list.txt --hcp
Loading organisms names success.....


The result are store in:Outdata


Now loading data and constructing phylogenetic tree......
2016-10-29 19:44:11,660 KEGG INDEX DB INFO: Read organisms names success
2016-10-29 19:44:17,296 KEGG INDEX DB INFO: Retrieve and download of highly conserved protein 'Ribosomal protein L1' was successful store in p1.fasta file
2016-10-29 19:44:17,919 KEGG INDEX DB INFO: Retrieve and download of highly conserved protein 'DNA-directed RNA polymerase subunit alpha' was successful store in p2.fasta file
2016-10-29 19:44:18,369 KEGG INDEX DB INFO: Retrieve and download of highly conserved protein 'Leucyl-tRNA synthetase' was successful store in p3.fasta file
2016-10-29 19:44:18,943 KEGG INDEX DB INFO: Retrieve and download of highly conserved protein 'Metal-dependent proteases with chaperone activity' was successful store in p4.fasta file
2016-10-29 19:44:19,660 KEGG INDEX DB INFO: Retrieve and download of highly conserved protein 'Phenylalanine-tRNA synthethase alpha subunit' was successful store in p5.fasta file
```

```
2016-10-29 19:44:20,114 KEGG INDEX DB INFO: Retrieve and download of highly conserved protein 'Predicted GTPase probable translation factor' was successful store in p6.fasta file

2016-10-29 19:44:20,505 KEGG INDEX DB INFO: Retrieve and download of highly conserved protein 'Ribosomal protein L11' was successful store in p7.fasta file

2016-10-29 19:44:20,917 KEGG INDEX DB INFO: Retrieve and download of highly conserved protein 'Ribosomal protein L13' was successful store in p8.fasta file

2016-10-29 19:44:21,333 KEGG INDEX DB INFO: Retrieve and download of highly conserved protein 'Ribosomal protein L14' was successful store in p9.fasta file

......
```

Outputs:

```
log.log

Outdata/

        RAxML_bestTree.T1

        RAxML_bipartitions.T1

        RAxML_bipartitionsBranchLabels.T1

        RAxML_bootstrap.T1

        RAxML_info.T1

   temp/

        conserved_protein20161029194411/

                                         p1.fasta

                                         p2.fasta

                                         p3.fasta

                                         ......

        alignment20161029194429/

                                    p1.fasta

                                    p2.fasta

                                    p2.fasta

                                    ......

        concatenate20161029194432/

                                  concatenate.fasta
```

```
                              concatenate.fasta-gb1

                              concatenate.fasta-gb1.htm

                              concatenate.fasta-gb1.phy
```

- `log.log`: logs.
- `Outdata`: tree files.
    - `RAxML_bestTree.T1`: best ML search tree built by RAxML.
    - `RAxML_bipartitions.T1`: bipartition tree built by RAxML.
    - `RAxML_bipartitionsBranchLabels.T1`: bipartition tree by RAxML with branch length.
    - `RAxML_bootstrap.T1`: bootstrap result.
    - `RAxML_info.T1`: logs in running RAxML.
- `temp`: temporary data used to check the quality of outputs in each step.
    - `conserved_protein`: highly conserved proteins retrieved from the KEGG database.
    - `alignment`: aligned sequences.
    - `concatenate`: concatenated sequences and conserved blocks.
        - `concatenate.fasta`: concatenated HCP sequences.
        - `concatenate.fasta-gb1`: conserved blocks (by Gblocks).
        - `concatenate.fasta-gb1.htm`: conserved blocks displayed in html.
        - `concatenate.fasta-gb1.phy`: conserved blocks in the PHYLIP format.

Automatically reconstruct species trees by SSU rRNA

```
$ PhySpeTree autobuild -i organism_example_list.txt --srna
```

```
Loading organisms names success.....


The result are store in:Outdata


Now loading data and constructing phylogenetic tree......

2016-10-29 20:12:49,353 SSU rRNA DB INFO: Read organisms names success

2016-10-29 20:12:54,582 SSU rRNA DB INFO: Retrieve and download of organism 'a
ca' SSU rRNA sequence was successful

2016-10-29 20:12:56,831 SSU rRNA DB INFO: Retrieve and download of organism 'a
ce' SSU rRNA sequence was successful

2016-10-29 20:12:59,182 SSU rRNA DB INFO: Retrieve and download of organism 'a
cl' SSU rRNA sequence was successful

2016-10-29 20:13:01,545 SSU rRNA DB INFO: Retrieve and download of organism 'a
cn' SSU rRNA sequence was successful

2016-10-29 20:13:04,096 SSU rRNA DB INFO: Retrieve and download of organism 'a
co' SSU rRNA sequence was successful

2016-10-29 20:13:06,972 SSU rRNA DB INFO: Retrieve and download of organism 'a
cp' SSU rRNA sequence was successful

2016-10-29 20:13:09,943 SSU rRNA DB INFO: Retrieve and download of organism 'a
dg' SSU rRNA sequence was successful

2016-10-29 20:13:12,707 SSU rRNA DB INFO: Retrieve and download of organism 'a
dk' SSU rRNA sequence was successful

2016-10-29 20:13:16,015 SSU rRNA DB INFO: Retrieve and download of organism 'a
eh' SSU rRNA sequence was successful

2016-10-29 20:13:18,969 SSU rRNA DB INFO: Retrieve and download of organism 'a
eq' SSU rRNA sequence was successful
```

Outputs:

```
log.log

Outdata/

        RAxML_bestTree.T1

        RAxML_bipartitions.T1

        RAxML_bipartitionsBranchLabels.T1
```

```
        RAxML_bootstrap.T1

        RAxML_info.T1

   temp/

        rna_sequence20161029201249/

                                  rna_sequence.fasta


        rna_alignment20161029201319/

                                   rna_sequence.fasta

                                   rna_sequence.fasta-gb1

                                   rna_sequence.fasta-gb1.htm

                                   rna_sequence.fasta-gb1.phy
```

- `log.log`: logs.
- `Outdata`: tree files like the HCP method.
- `temp`: temporary data used to check the quality of outputs in each step.
    - `rna_sequence`: SSU rRNA sequences retrieved from the SILVA database.
    - `rna_alignment`: aligned sequences and conserved blocks.
        - rna_sequence.fasta: aligned SSU rRNA sequences.
        - rna_sequence.fasta-gb1: conserved blocks (by Gblocks).
        - rna_sequence.fasta-gb1.htm: conserved blocks displayed in html.
        - rna_sequence.fasta-gb1.phy: conserved blocks in the PHYLIP format.

Advanced options

Advanced options of internal software called in PhySpeTree can be set. These options are `enclosed in single quotes and start with a space`.

Here is an example of setting RAxML advanced options by `--raxml_p`:

```
$ PhySpeTree autobuild -i organism_example_list.txt -o test --srna --raxml --raxml_p ' -f a -m GTRGAMMA  -p 12345 -x 12345 -# 100 -n T1'
```

--muscle

Multiple sequence alignment by MUSCLE (default).
--muscle_p

Set MUSCLE advanced parameters, please see MUSCLE Manual
The default option:

option	description
-maxiter	Maximum number of iterations to run. The default is 100.

--clustalw

Multiple sequence alignment by ClustalW2.
--clustalw_p

Set ClustalW2 advanced parameters, please see Clustalw Help.
--mafft

Multiple sequence alignment by mafft.
--mafft_p

Set mafft advance parameters. Here use mafft default parameters, please see mafft algorithms
--gblocks

Trim by Gblocks.(default)
--gblocks_p

Set Gblocks advanced parameters, please see Gblocks documentation.
The default option:

option	description
-t	Choice type of sequence (default).
-e	Generic file extension. The default in PhySpeTree is "-gbl1".

--trimal

Trim by trimal.
--trimal_p

Set trimal advance parameters, please seetrimal command line

--ranxml

Reconstruct species tree by RAxML (default).

--raxml_p

Set RAxML advanced parameters, please see RAxML Manual.

The default option:

option	description
-f	select algorithm. The default in PhySpeTree is `a`, rapid Bootstrap analysis and search for bestsco program run.
-m	Model of binary (morphological), nucleotide, multiState, or amino acid substitution. The PhySpeTre PROTGAMMAJTTX.
-p	Specify a random number seed for the parsimony inferences. The default in PhySpeTree is 12345
-x	Specify an integer number (random seed) and turn on rapid bootstrapping. The default in PhySpeT
-N	The same with -# specify the number of alternative runs on distinct starting trees. The default in Ph

--fasttree

Reconstruct species tree by FastTree.

--fasttree_p

Set FastTree advanced parameters, please see FastTree Helps.

--iqtree

Reconstruct species tree by iqtree.

--iqtree_p

Set iqtree advanced parameters, please see IQ-TREE.

build

The `build` module is used to reconstruct species trees with manually prepared sequences. Advanced options are the same as `autobuild` module.

```
# multiple method

$ PhySpeTree build -i example_hcp -o output --multiple


# single method

$ PhySpeTree build -i example_16s_ssurna.fasta -o output --single
```

build options

option	Description
-h	Print help message and exits.
-i	Input a TXT file containing abbreviated species names.
-o	A directory to store outputs. The default is "Outdata".
-t	Number of processing threads (CPUs). The default is 1.
--multiple	Specify concatenate highly conserved protein method to reconstruct phylogenetic tree.
--single	Use SSU rRNA data to reconstruct phylogenetic tree.

Example

Build species trees by manually prepared HCP

The HCP sequences belonging to the same class are prepared in one FASTA format file, and all FASTA format files are stored in the same folder. For example, the folder example_build_hcp contains 10 classes of HCP (p1~p10) corresponding to 10 different organisms. There is no limit number of HCP sequences. We recommend no less than 10 highly conserved proteins to ensure the accuracy of the reconstructed phylogenetic tree.

Download and decompress the example input file:

```
$ wget "https://yangfangs.github.io/physpetools/example/example_build_hcp.tar.gz"

--2016-10-29 20:40:41--  https://yangfangs.github.io/physpetools/example/example_build_hcp.tar.gz
Resolving yangfangs.github.io (yangfangs.github.io)... 151.101.48.133
Connecting to yangfangs.github.io (yangfangs.github.io)|151.101.48.133|:443... connected.
HTTP request sent, awaiting response... 200 OK
Length: 17419 (17K) [application/octet-stream]
Saving to: ‘example_build_hcp.tar.gz’

example_build_hcp.tar.gz    100%[===============================================>]  17.01K  --.-KB/s    in 0.009s

2016-10-29 20:40:42 (1.92 MB/s) - ‘example_build_hcp.tar.gz’ saved [17419/17419]


$ tar -zxvf example_build_hcp.tar.gz

example_build_hcp/

example_build_hcp/p1.fasta
example_build_hcp/p2.fasta
example_build_hcp/p3.fasta
example_build_hcp/p4.fasta
example_build_hcp/p5.fasta
example_build_hcp/p6.fasta
example_build_hcp/p7.fasta
example_build_hcp/p8.fasta
```

```
example_build_hcp/p9.fasta

example_build_hcp/p10.fasta
```

Check HCP:

```
$ cd  example_build_hcp/

$ cat p1.fasta

>aeh

MARLTKRQKAIREKIDPAQQYPVAEALGLLRELPGAKFTESVEVAVNLGVDPRKSDQIVR

GSTVLPNGTGKTVRVAVFAQGDAAEAAKEAGADIVGMDDLAEQVKGGNLDFDVVVAAPDA

MGVVGRLGPILGPRGLMPNPKVGTVTPDVAGAVKNAKAGQVRYRTDKGGIIHCAIGKVDF

EVEALQQNLQALITDLQKLKPANSKGVYLKKVAVSTTMGPGLAVDLASLET

>adk

MAKLTKKQKAQQGKVDSTKLYPFAEAVALVKEAATAKFDESIDVAVQLGVDAKKSDQVVR

GAVVLPNGTGKTTRVAVFAQGAKAEEAKAAGADVVGMDDLAAQVKAGDMPFDVVIAAPDA

MRVVGTLGQILGPRGLMPNPKVGTVTPDVATAVKNAKAGQVQFRVDKAGIVHTTIGRRSF

ADDKLQGNLAALIEALNKAKPATSKGVYLRKVAVSSTMGVGVRVDTQSIAA

>acp

MAHVAKKYKAAAEKVDRTKRYKLDEAMSLVKQTATKKFDETVDASINLGVDPKHADQVVR

GAVVLPHGMGKTVRLAVFAKGDKAKEAQEAGADIVGAEDLAEKIQGGFMDFDKLIATPDM

MGVVGRLGKILGPRGLMPNPKVGTVTMDLARAVKEQKAGKVEFRVEKAGIVHVPFGKASF

DPDKLKANFSAIMEVIYKAKPQTAKGVYVKNVTLSTTMGPGIKVDLAELAAQHA

>acn

MSGDGSSYSAEEGIRELLQSAKAKFRESVDVAIKLSVADSKSGESVRGAVVLPKGLGREV

RVAVFAKGEHAKHASDAGADVVGDEELIEEIKKGRKLDVDWCIATPDFMPQISAIAKILG

PRGLMPNPKFGTVTLELAKMVGVIKSGQVKFKSDRYGIVHVKIGDVSFTPEDLLENFNAV

VVAVQNLKPATIKGSYVRGVFVNSTMGRSFRIAGIG

>adg

MPKHGKKYLEAKKQVDRTKLYDPYEALELVKRLASAKFDETVEVAVRLGVDPRHADQQVR

GAVVLPHGTGKTRRVLVFARGEKAKEAEAAGADYVGAEDLIARIQGGWLDFDVAIATPDM

MAMVGRIGRILGPRGLMPNPKTGTVTFDVAQAVAEAKAGRVEYRTDKAGIVHAPIGKVSF
```

```
EVEKLVENLKALVDALVRAKPPAAKGQYLRSITVSSTMGPGVKVNPAKLLAS

>acl

MKRGKKYLEAVKLYDKSVAYTGLEAVELAKKTSVAKFDATVEVAFRLNVDPRKADQNLRG

AISLPHGTGKTVRVVVIAKPEKAKEALAAGALEAGDVELIDKIGKGWFDFDVMVATPDMM

AQLGKLGRVLGPKGLMPNPKTGTVTLDVAKAVEEIKAGKIEYRTDKVGNIHAPIGKVSFD

SNKLHENMLAIYNQLVRIKPATVKGTYIKKIALSTTMGPGIMVEENNIKK

>ace

MKRGKKYRAAAQLVDRTKLYSPLEAMRLAKQTNTMRVPATVEVAMRLGVDPRKADQMVRG

TVNLPHGTGKTPRVLVFATAERAEEARAAGADYVGADELIEQVANGFLDFDAVVATPDLM

GKVGRLGRILGPRGLMPNPKTGTVTNDVAKAVADIKSGKIEFRVDRQANLHLVIGKTDFT

EQQLVENYAAALDEVLRLKPPTAKGRYLKKVTISTTMGPGIPVDPNRVRNLLAEETAAA

>aeq

MTKHGKKYVEAEKQIPAEPVSPLAAMKLLKEISVANFDETVTGDFRLGIDTRQADQQLRG

TVSLPNGSGKTVRVAVFAEGAAAQAAEEAGADIVGTDELMQQIQAGEFNFDAAVATPDQM

GKVGRLGKILGPRGLMPNPKLGTVTNDVAKAIKELKGGRVEYRADRYGIAHVVLGKVSFT

PEQLAENYGAVYDEILRMKPAAAKGKYVKSITVSGTMTPGVSVDSSVTRAYTESAE

>aca

MSKKVSKNVAKARAAVEPRPYTLQDAVPLLQQVKFAKFDETVDLTMRLGVDPRHADQMVR

GTVVLPHGLGKTKKVAVITTGDRQKEAEAAGAEIVGGEELVEKIQKESWTDFDALIATPD

MMRSVGRLGKVLGPRGLMPNPKTGTVTNDVAAAVKEIKAGKIEYRTDKTALVHVPVGKLS

FPAEKLIDNAMTVITSVVRAKPSAAKGKYIKGITLSSTMGPGIPLDGSVADAAAKA

>aco

MAKKSKRYSEIAAKVDSTKLYGLREAVDLYKEVATAKFDESLEVHIRLGVDPRHADQQVR

GTIVLPHGTGITKRVLVLAVGEKVKEAEDAGADIVGGDDLIQKISTGWLDFDAVIATPDM

MKSVGRLGKILGPRGLMPSAKAGTVTFDVADAIKEIKAGRVEFRVDKTAIIHNMVGKKSF

EAEKLFENLKVLYRAILKARPASAKGTYVRSFYIAPTMGVGIKIDPVAASKEVAEA
```

Reconstruct species tree and store outputs in the `build_hcp_tree` folder:

```
PhySpeTree build -i example_build_hcp -o build_hcp_tree --multiple
```

Build species trees by manually prepared SSU rRNA

All SSU rRNA sequences are prepared in one FASTA format file, for example example_build_srna.

Download and decompress the example input file:

```
$ wget "https://yangfangs.github.io/physpetools/example/example_build_srna.fasta"


--2016-10-29 20:56:31--  https://yangfangs.github.io/physpetools/example/example_build_srna.fasta

Resolving yangfangs.github.io (yangfangs.github.io)... 151.101.48.133

Connecting to yangfangs.github.io (yangfangs.github.io)|151.101.48.133|:443... connected.

HTTP request sent, awaiting response... 200 OK

Length: 14982 (15K) [application/octet-stream]

Saving to: ‘example_build_srna.fasta’


example_build_srna.fasta     100%[===============================================>]  14.63K  --.-KB/s    in 0.005s


2016-10-29 20:56:33 (3.14 MB/s) - ‘example_build_srna.fasta’ saved [14982/14982]


$ grep '>' example_build_srna.fasta

>aca

>ace

>acl

>acn

>aco

>acp

>adg

>adk

>aeh
```

```
>aeq
```

Reconstruct species tree and store outputs in the `build_srna_tree` folder:

```
PhySpeTree build -i example_build_srna.fasta -o build_srna_tree --single --fas
ttree
```

combine

The **combine** module is used to combine trees generated from different methods. It contains two steps, at first merge different tree files into the same file. You can use `cat` bash command in the Linux system, for example:

```
$ cat tree1.tree tree2.tree > combineTree.tree
```

Then, use **combine**:

```
$ PhySpeTree combine -i combineTree.tree [options]*
```

combine options

option	Description
-h	Print help message and exits.
-i	Input PHYLIP format file containing multiple trees.
-o	Output directory. The default is "combineTree".
--mr	Majority rule trees.
--mre	Extended majority rule trees.
--strict	Strict consensus trees.

option	Description
--astral	Use ASTRAL combine multi gene tree.
--supertree	Use Spr_Supertree combining conflicting evolutionary histories that are due to lateral gene tr

Example

example_combine_tree.tar.gz contains `tree1.tree` and `tree2.tree` reconstructed by the HCP and SSU rRNA method, respectively.

Download and decompress the example input file:

```
$ wget "https://yangfangs.github.io/physpetools/example/example_combine_tree.tar.gz"


--2016-10-30 13:32:06--  https://yangfangs.github.io/physpetools/example/example_combine_tree.tar.gz
Resolving yangfangs.github.io (yangfangs.github.io)... 151.101.48.133
Connecting to yangfangs.github.io (yangfangs.github.io)|151.101.48.133|:443... connected.
HTTP request sent, awaiting response... 200 OK
Length: 661 [application/octet-stream]
Saving to: ‘example_combine_tree.tar.gz’


example_combine_tree.tar.gz  100%[=============================================>]     661  --.-KB/s    in 0s


2016-10-30 13:32:07 (380 MB/s) - ‘example_combine_tree.tar.gz’ saved [661/661]
```

```
$ tar -zxvf example_combine_tree.tar.gz

example_combine_tree/

example_combine_tree/tree2.tree

example_combine_tree/tree1.tree
```

Merge `tree1.tree` and `tree2.tree`:

```
$ cd example_combine_tree/

$ cat tree1.tree tree2.tree > combine.tree
```

Combine trees:

```
PhySpeTree combine -i combine.tree -o combineTree
```

Outputs:

```
combine/

       RAxML_info.T1

       RAxML_MajorityRuleConsensusTree.T1
```

- `RAxML_info.T1`: logs in running RAxML.
- `RAxML_MajorityRuleConsensusTree.T1`: the majority rule consensus tree.

Using `--astral` option

Notice: The --astral option calls the third-party software ASTRAL. Please be aware that JRE has been installed in your running environments. For users who run the Docker image of PhySpeTree, JRE is unnecessary.

```
PhySpeTree combine -i combine.tree -o combineTree --astral
```

Outputs:

```
combineTree/

          combine.tree
```

Using `--supertree` option

- Use Spr_Supertree combining conflicting evolutionary histories that are due to lateral gene transfer (LGT).

Download example tree:

```
$ wget "https://yangfangs.github.io/physpetools/example/trees.tree"

--2019-10-02 15:49:42--  https://yangfangs.github.io/physpetools/example/tree
s.tree

Resolving yangfangs.github.io (yangfangs.github.io)... 185.199.108.153, 185.1
99.109.153, 185.199.110.153, ...

Connecting yangfangs.github.io (yangfangs.github.io)|185.199.108.153|:443...
connected.

HTTP request sent, awaiting response... 200 OK

Length: 661 [application/octet-stream]

Saving to: “trees.tree”



trees.tree              100%[======================================>]     140
--.-KB/s  用时 0s



2019-10-02 15:49:44 (168 MB/s) - saved “trees.tree” [140/140])
```

`supertree` option:

```
PhySpeTree combine -i trees.tree -o Supertree --supertree
```

Outputs:

```
Supertree/

        spr_supertree.tree
```

iview

PhySpeTree provides the `iview` module to annotate taxonomic information (kingdom, phylum, class, or order) of output trees and to generate configure files linked to iTol.

```
$ PhySpeTree iview -i organism_example_list.txt --range
```

iview options

option	Description
-h	Print help message and exits.
-i	Input a TXT file containing abbreviated species names.
-o	A directory to store outputs. The default is "iview".
-a	Colored ranges [kingdom, phylum, class or order].
-r/--range	Annotating labels with ranges by kingdom, phylum, class or order. The default is phylum.
-c/--color	Annotating labels without ranges by kingdom, phylum, class or order. The default is phylum.
-l/--labels	Change species labels from abbreviated names to full names.

Example

Download the example file:

```
$ wget "https://yangfangs.github.io/physpetools/example/organism_example_lis
t.txt"



--2016-10-30 13:40:48--  https://yangfangs.github.io/physpetools/example/orga
nism_example_list.txt

Resolving yangfangs.github.io (yangfangs.github.io)... 151.101.48.133
```

```
Connecting to yangfangs.github.io (yangfangs.github.io)|151.101.48.133|:443... connected.

HTTP request sent, awaiting response... 200 OK

Length: 39 [text/plain]

Saving to: ‘organism_example_list.txt’



organism_example_list.txt    100%[==============================================>]      39  --.-KB/s    in 0s



2016-10-30 13:40:50 (21.5 MB/s) - ‘organism_example_list.txt’ saved [39/39]
```

Annotate the tree by kingdom

```
$ PhySpeTree iview -i organism_example_list.txt --range -a kingdom

Color range by kingdom was complete.
```

The color range file is store in the `iview` folder:

```
$ cd iview

$ cat range_color_by_kingdom.txt

TREE_COLORS

SEPARATOR TAB

DATA

aca     range   #BEBF5A Prokaryotes

ace     range   #BEBF5A Prokaryotes

acl     range   #BEBF5A Prokaryotes

acn     range   #BEBF5A Prokaryotes

aco     range   #BEBF5A Prokaryotes

acp     range   #BEBF5A Prokaryotes

adg     range   #BEBF5A Prokaryotes

adk     range   #BEBF5A Prokaryotes

aeh     range   #BEBF5A Prokaryotes
```

```
aeq	range	#BEBF5A Prokaryotes
```

Annotate the tree by phylum

```
$ PhySpeTree iview -i organism_example_list.txt --range -a phylum

Color range by phylum was complete.
```

The color range file is store in the `iview` folder:

```
$ cd iview

$ cat range_color_by_phylum.txt

TREE_COLORS

SEPARATOR TAB

DATA

aca	range	#865142 Bacteria

ace	range	#865142 Bacteria

acl	range	#865142 Bacteria

acn	range	#865142 Bacteria

aco	range	#865142 Bacteria

acp	range	#865142 Bacteria

adg	range	#865142 Bacteria

adk	range	#865142 Bacteria

aeh	range	#865142 Bacteria

aeq	range	#865142 Bacteria
```

Annotate the tree by class

```
$ PhySpeTree iview -i organism_example_list.txt --range -a class

Color range by class was complete.
```

The color range file is store in the `iview` folder:

```
$ cd iview

$ cat range_color_by_class.txt
```

```
TREE_COLORS
SEPARATOR TAB
DATA
aca	range	#9AB7F3 Acidobacteria
ace	range	#99D1DB Actinobacteria
acl	range	#A5E58D Tenericutes
acn	range	#94F1C1 Alphaproteobacteria
aco	range	#D67A21 Synergistetes
acp	range	#DD9284 Deltaproteobacteria
adg	range	#3E70B8 Firmicutes - Clostridia
adk	range	#DDC8B7 Betaproteobacteria
aeh	range	#72E137 Gammaproteobacteria - Others
aeq	range	#99D1DB Actinobacteria
```

Annotate the tree by order

```
$ PhySpeTree iview -i organism_example_list.txt --range -a order
Color range by order was complete.
```

The color range file is store in the `iview` folder:

```
$ cd iview
$ cat range_color_by_order.txt
TREE_COLORS
SEPARATOR TAB
DATA
aca	range	#AA8761 Acidobacterium
ace	range	#8770BC Acidothermus
acl	range	#3BD26B Acholeplasma
acn	range	#D1B487 Anaplasma
aco	range	#D96D21 Aminobacterium
acp	range	#AC4E16 Anaeromyxobacter
```

```
adg     range   #287AD8 Ammonifex

adk     range   #C8184E Alicycliphilus

aeh     range   #57A569 Alkalilimnicola

aeq     range   #F1A2B7 Adlercreutzia
```

check

The `check` module is used to check whether input organisms are in pre-built databases.

```
$ PhySpeTree check -i organism_example_list.txt --ehcp
```

check options

option	Description
-h	Print help message and exits.
-i	Input a TXT file containing abbreviated species names.
-o	A directory to store outputs. The default is "check".
--hcp	Check whether organisms are supported in the KEGG database.
--ehcp	Check input organisms prepare for extend autobuild tree module.
--srna	Check whether organisms are supported in the SILVA database.

Example

Check extended organisms in `autobuild`

Determine proteins to be prepared in the `autobuild` module with the `--ehcp` option, for example, organism_example_list.txt

Download the example file:

```
$ wget "https://yangfangs.github.io/physpetools/example/organism_example_lis
t.txt"


--2016-10-30 13:40:48--  https://yangfangs.github.io/physpetools/example/orga
nism_example_list.txt

Resolving yangfangs.github.io (yangfangs.github.io)... 151.101.48.133

Connecting to yangfangs.github.io (yangfangs.github.io)|151.101.48.133|:44
3... connected.

HTTP request sent, awaiting response... 200 OK

Length: 39 [text/plain]

Saving to: ‘organism_example_list.txt’


organism_example_list.txt    100%[==========================================
===>]      39  --.-KB/s    in 0s


2016-10-30 13:40:50 (21.5 MB/s) - ‘organism_example_list.txt’ saved [39/39]
```

Check:

```
$ PhySpeTree check -i organism_example_list.txt --ehcp


'Ribosomal protein L1' -----------------------------------> p1.fasta


'DNA-directed RNA polymerase subunit alpha' ----------------------------------
-> p2.fasta


'Leucyl-tRNA synthetase' -----------------------------------> p3.fasta


'Metal-dependent proteases with chaperone activity' --------------------------
---------> p4.fasta
```

```
'Phenylalanine-tRNA synthethase alpha subunit' ------------------------------
----> p5.fasta

'Predicted GTPase probable translation factor' ------------------------------
----> p6.fasta

'Ribosomal protein L11' ----------------------------------> p7.fasta

'Ribosomal protein L13' ----------------------------------> p8.fasta

'Ribosomal protein L14' ----------------------------------> p9.fasta

'Ribosomal protein L22' ----------------------------------> p10.fasta

'Ribosomal protein L3' ----------------------------------> p11.fasta

'Ribosomal protein L5' ----------------------------------> p12.fasta

'Ribosomal protein S11' ----------------------------------> p13.fasta

'Ribosomal protein S17' ----------------------------------> p14.fasta

'Ribosomal protein S2' ----------------------------------> p15.fasta

'Ribosomal protein S3' ----------------------------------> p16.fasta

'Ribosomal protein S4' ----------------------------------> p17.fasta

'Ribosomal protein S5' ----------------------------------> p18.fasta
```

```
'Ribosomal protein S7' -----------------------------------> p19.fasta

'Ribosomal protein S8' -----------------------------------> p20.fasta

'Ribosomal protein S9' -----------------------------------> p21.fasta

'Seryl-tRNA synthetase' -----------------------------------> p22.fasta

'Arginyl-tRNA synthetase' -----------------------------------> p23.fasta

'DNA-directed RNA polymerase beta subunit' ---------------------------------
-> p24.fasta

'Ribosomal protein S13' -----------------------------------> p25.fasta

Check extend highly conserved protein is completed.
```

The check result is stored in the `check` folder. In `physpe_echp_extend.txt` file indicates class of HCP and their corresponding names, which will be used to prepare extended HCP sequences.

```
$ cd check

$ cat physpe_echp_extend.txt

'Ribosomal protein L1' -----------------------------------> p1.fasta

'DNA-directed RNA polymerase subunit alpha' ---------------------------------
-> p2.fasta

'Leucyl-tRNA synthetase' -----------------------------------> p3.fasta

'Metal-dependent proteases with chaperone activity' -------------------------
---------> p4.fasta

'Phenylalanine-tRNA synthethase alpha subunit' -----------------------------
----> p5.fasta
```

```
'Predicted GTPase probable translation factor' ----------------------------------> p6.fasta
'Ribosomal protein L11' ----------------------------------> p7.fasta
'Ribosomal protein L13' ----------------------------------> p8.fasta
'Ribosomal protein L14' ----------------------------------> p9.fasta
'Ribosomal protein L22' ----------------------------------> p10.fasta
'Ribosomal protein L3' ----------------------------------> p11.fasta
'Ribosomal protein L5' ----------------------------------> p12.fasta
'Ribosomal protein S11' ----------------------------------> p13.fasta
'Ribosomal protein S17' ----------------------------------> p14.fasta
'Ribosomal protein S2' ----------------------------------> p15.fasta
'Ribosomal protein S3' ----------------------------------> p16.fasta
'Ribosomal protein S4' ----------------------------------> p17.fasta
'Ribosomal protein S5' ----------------------------------> p18.fasta
'Ribosomal protein S7' ----------------------------------> p19.fasta
'Ribosomal protein S8' ----------------------------------> p20.fasta
'Ribosomal protein S9' ----------------------------------> p21.fasta
'Seryl-tRNA synthetase' ----------------------------------> p22.fasta
'Arginyl-tRNA synthetase' ----------------------------------> p23.fasta
'DNA-directed RNA polymerase beta subunit' ----------------------------------> p24.fasta
'Ribosomal protein S13' ----------------------------------> p25.fasta
```

Check whether input organisms are supported in PhySpeTree

Check whether input species are supported by the KEGG database when using the `--hcp` method, for example example download.

Download the example file:

```
$ wget "https://yangfangs.github.io/physpetools/example/191speciesnames.txt"
```

```
--2016-10-30 14:48:21--  https://yangfangs.github.io/physpetools/example/191speciesnames.txt

Resolving yangfangs.github.io (yangfangs.github.io)... 151.101.48.133

Connecting to yangfangs.github.io (yangfangs.github.io)|151.101.48.133|:443... connected.

HTTP request sent, awaiting response... 200 OK

Length: 773 [text/plain]

Saving to: ‘191speciesnames.txt’



191speciesnames.txt         100%[=============================================>]     773  --.-KB/s    in 0s



2016-10-30 14:48:22 (322 MB/s) - ‘191speciesnames.txt’ saved [773/773]
```

The check results show one organism named 'ges' is not supported in PhySpeTree:

```
$ PhySpeTree check -i 191speciesnames.txt --hcp

WARNING: The following species are not supported by KEGG DATABASE:

ges

Checked  whether the input species names in KEGG DATABASE completed.
```

Check whether input species are supported by SILVA database when using the `--srna` metho, for example [example download]()

Download the example file:

```
$ wget "https://yangfangs.github.io/physpetools/example/191speciesnames.txt"



--2016-10-30 14:48:21--  https://yangfangs.github.io/physpetools/example/191speciesnames.txt

Resolving yangfangs.github.io (yangfangs.github.io)... 151.101.48.133

Connecting to yangfangs.github.io (yangfangs.github.io)|151.101.48.133|:443... connected.

HTTP request sent, awaiting response... 200 OK
```

```
Length: 773 [text/plain]

Saving to: ‘191speciesnames.txt’



191speciesnames.txt         100%[============================================
==>]     773  --.-KB/s    in 0s



2016-10-30 14:48:22 (322 MB/s) - ‘191speciesnames.txt’ saved [773/773]
```

The check results show 28 organisms are not supported in PhySpeTree:

```


(progect) [yangfang@localhost test_check] $ PhySpeTree check -i 191speciesname
s.txt --srna

WARNING: The following species are not supported by SILVA DATABASE:

neq

ape

tac

mmp

gla

tps

cho

ddi

spo

aga

tru

mpu

lin

ban

bce

ljo

san

spg
```

```
ges

lis

sco

cdi

mle

wsu

rpr

bpe

bpa

ppr

Checked  whether the input species names in SILVA DATABASE completed.
```

For organisms not in the pre-built list, PhySpeTree provides extend options (`--echp` or `--esrna`) to insert manually prepared sequences.

PhySpeTree Tutorial

Automatically reconstruct the tree-of-life

Here we show how to use PhySpeTree to reconstruct tree-of-life (191 organisms) by two methods (HCP and SSU rRNA).

Reconstruct the tree-of-life by the HCP method

Concept

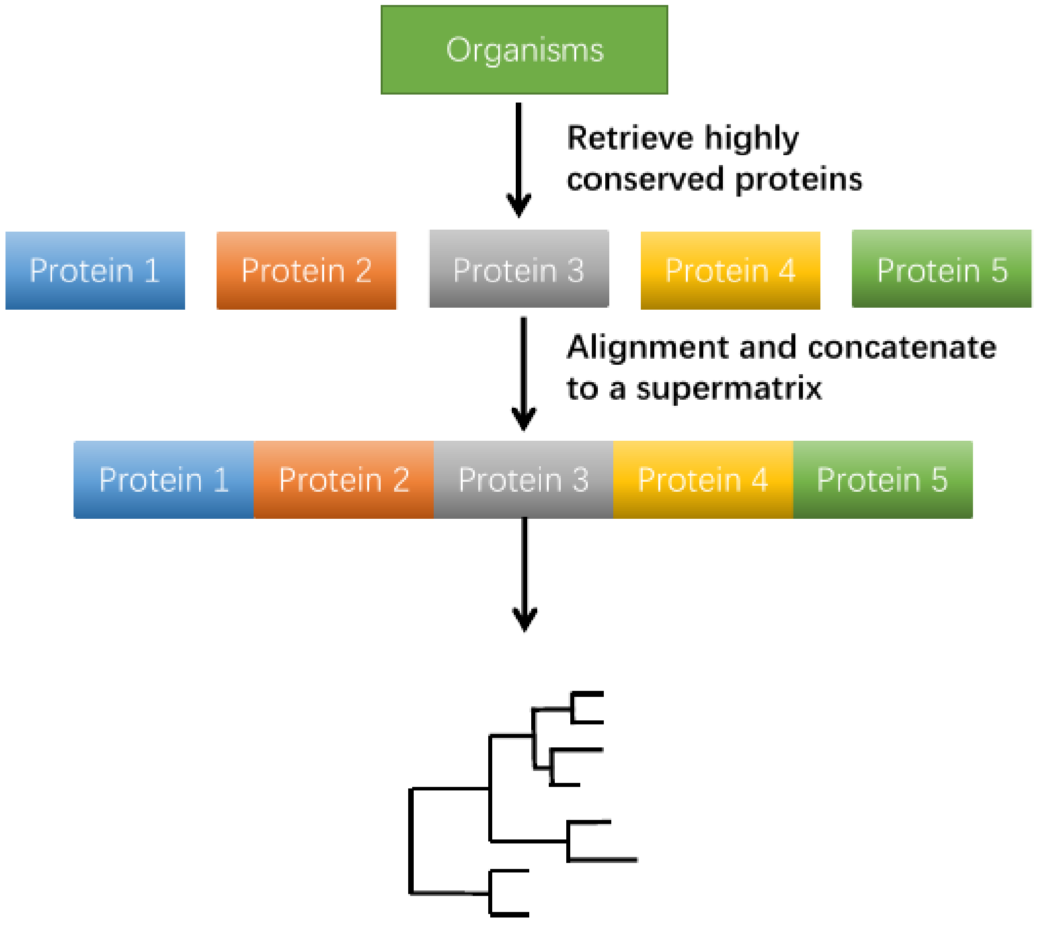

The main concept of the HCP method is to concatenate highly conserved proteins into a supermatrix and then build species tree. Here we prepared 31 classes of HCP to build the tree.

1. Prepare input species

Download 191 KEGG abbreviated species names:

```
$ wget "https://yangfangs.github.io/physpetools/example/191speciesnames.txt"


--2016-10-30 15:26:06--  https://yangfangs.github.io/physpetools/example/191s
peciesnames.txt

Resolving yangfangs.github.io (yangfangs.github.io)... 151.101.48.133

Connecting to yangfangs.github.io (yangfangs.github.io)|151.101.48.133|:44
3... connected.

HTTP request sent, awaiting response... 200 OK

Length: 773 [text/plain]
```

```
Saving to: ‘191speciesnames.txt’



191speciesnames.txt         100%[===========================================
==>]     773  --.-KB/s    in 0s



$ cat 191speciesnames.txt

neq

pai

ape

sto

ssoa

tvo

tac

afu

.....
```

2. Reconstruct the species tree

Use `--hcp` option to specify the HCP method.

```
$ PhySpeTree autobuild -i 191speciesnames.txt -o 191_pro --hcp



Loading organisms names success.....



The result are store in:191_pro



Now loading data and constructing phylogenetic tree......

2016-10-30 15:32:07,894 Checking organisms INFO: The species: ges can't match
in KEGG protein index database

2016-10-30 15:32:07,894 Checking organisms WARNING: These species can't match
in KEGG protein index database so removed and reconstruct phylogenetic tree.

2016-10-30 15:32:07,894 KEGG INDEX DB INFO: Read organisms names success
```

```
2016-10-30 15:32:34,213 KEGG INDEX DB INFO: Retrieve and download of highly co
nserved protein 'Ribosomal protein L1' was successful store in p1.fasta file

2016-10-30 15:33:00,679 KEGG INDEX DB INFO: Retrieve and download of highly co
nserved protein 'Leucyl-tRNA synthetase' was successful store in p2.fasta file

2016-10-30 15:33:25,052 KEGG INDEX DB INFO: Retrieve and download of highly co
nserved protein 'Ribosomal protein L14' was successful store in p3.fasta file

2016-10-30 15:33:49,649 KEGG INDEX DB INFO: Retrieve and download of highly co
nserved protein 'Ribosomal protein L5' was successful store in p4.fasta file

2016-10-30 15:34:14,510 KEGG INDEX DB INFO: Retrieve and download of highly co
nserved protein 'Ribosomal protein S7' was successful store in p5.fasta file

2016-10-30 15:34:41,123 KEGG INDEX DB INFO: Retrieve and download of highly co
nserved protein 'Arginyl-tRNA synthetase' was successful store in p6.fasta fil
e

2016-10-30 15:34:41,123 KEGG INDEX DB INFO: Retrieve from KEGG database 6 high
ly conserved proteins

......
```

3. Get the output tree

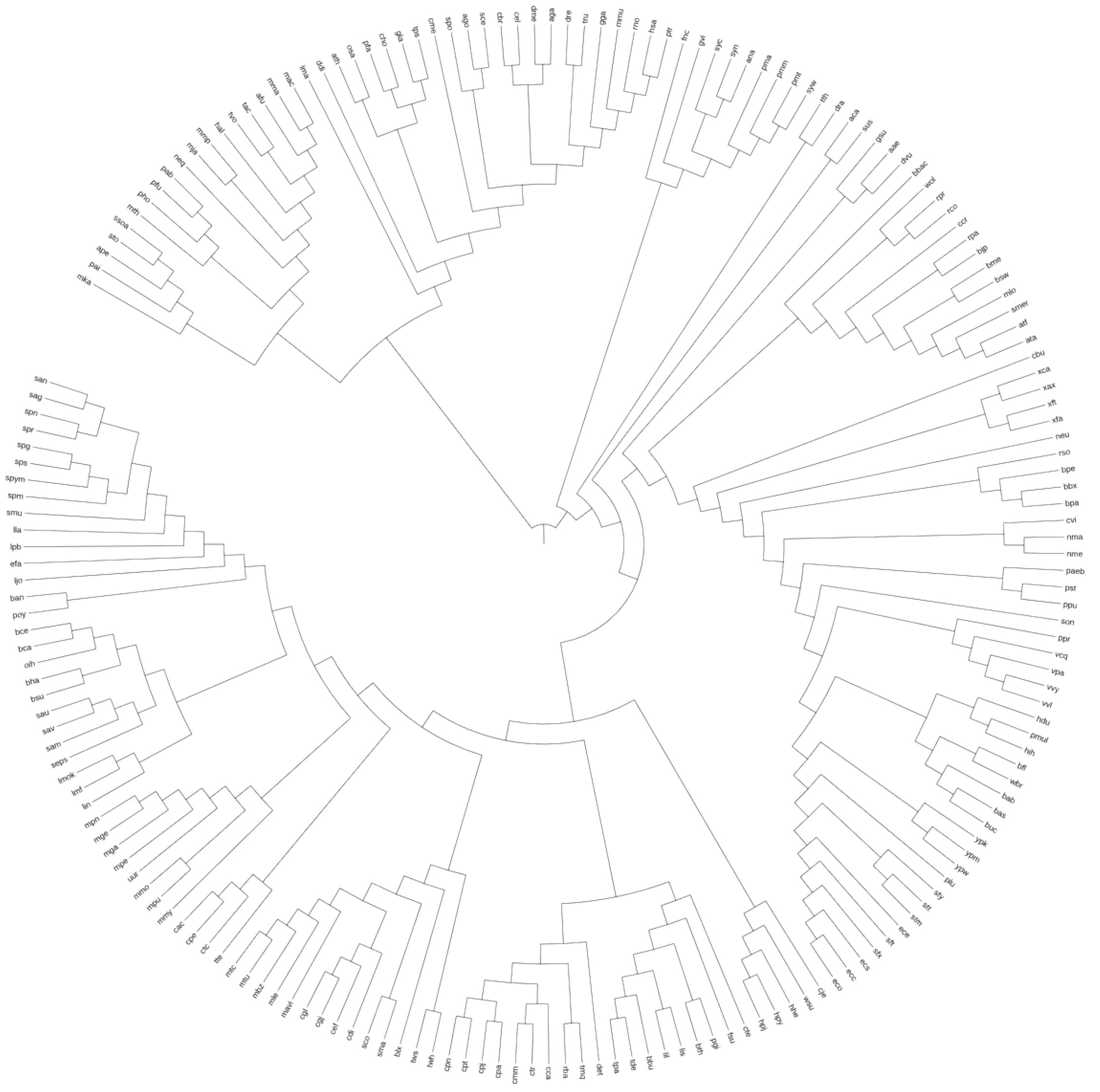

4. Annotate and view the tree

PhySpeTree provides the `iview` module to annotate trees with taxonomic information.

The output configure files can be directly used in iTOL, which is a very popular online tool for the display, annotation, and management of phylogenetic trees.

Use the `--labels` option to change abbreviated species names to full names:

```
$ PhySpeTree iview -i 191speciesnames.txt --labels

Change abbreviation names to full names complete

change labels file was save in iview/labels.txt
```

```
$ cd iview


$ cat labels.txt
LABELS
SEPARATOR TAB
DATA
neq	Nanoarchaeum equitans
pai	Pyrobaculum aerophilum
ape	Aeropyrum pernix
sto	Sulfolobus tokodaii
ssoa	Sulfolobus solfataricus SULA
tvo	Thermoplasma volcanium
tac	Thermoplasma acidophilum
afu	Archaeoglobus fulgidus DSM 4304
hal	Halobacterium sp. NRC-1
mac	Methanosarcina acetivorans
mma	Methanosarcina mazei Go1
pfu	Pyrococcus furiosus DSM 3638
pho	Pyrococcus horikoshii
pab	Pyrococcus abyssi
mth	Methanothermobacter thermautotrophicus
mka	Methanopyrus kandleri
mmp	Methanococcus maripaludis S2
.....
```

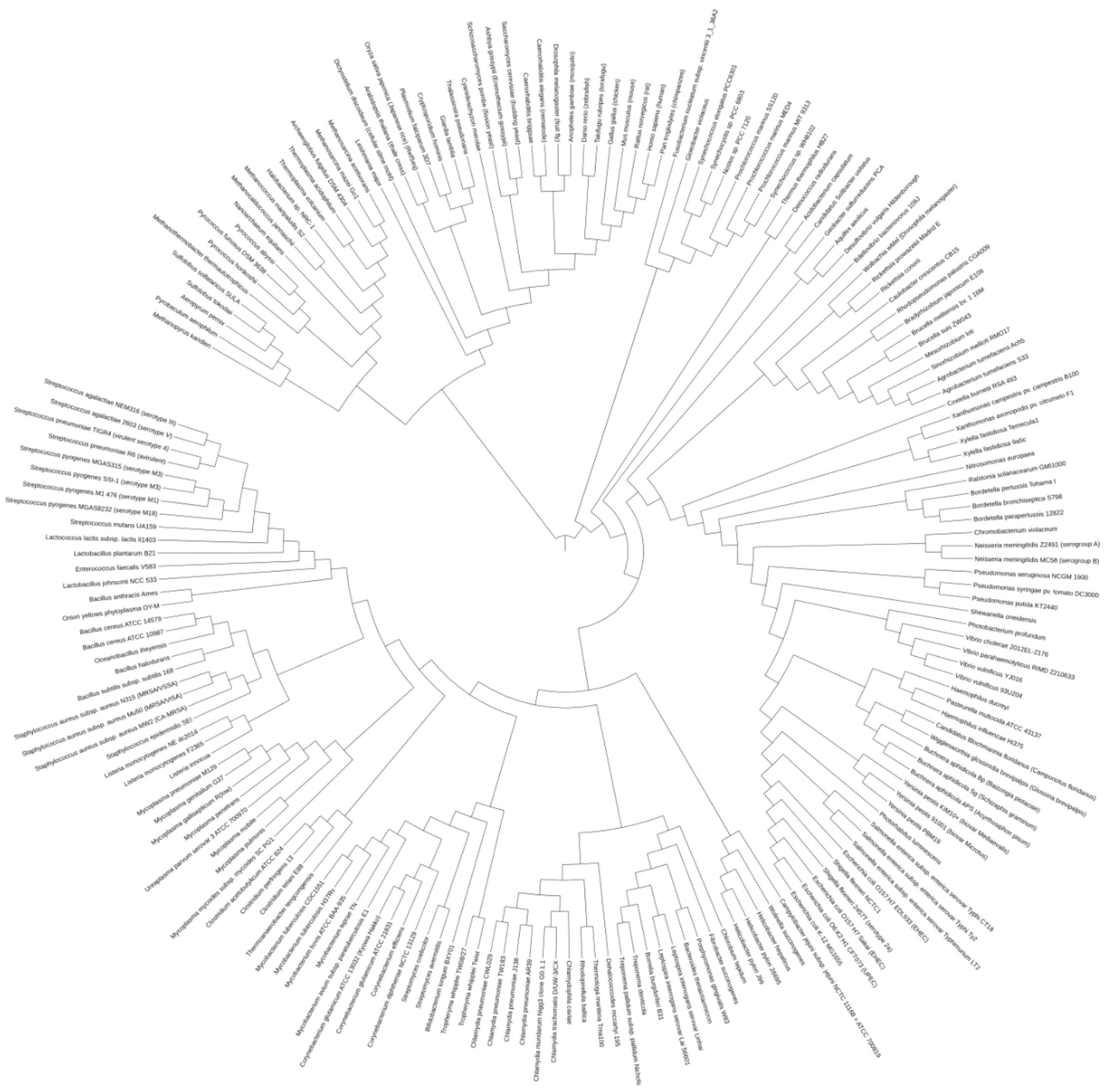

- Use the `-a` option to color branch ranges by phylum (default):

```
$ PhySpeTree iview -i 191speciesnames.txt -o iview --range -a phylum

Color range by phylum was complete.

Color range annotation was save in iview/range_color_by_phylum.txt



$ cd iview



$ cat range_color_by_phylum.txt

TREE_COLORS

SEPARATOR TAB
```

```
DATA
neq	range	#996433 Archaea
pai	range	#996433 Archaea
ape	range	#996433 Archaea
sto	range	#996433 Archaea
ssoa	range	#996433 Archaea
tvo	range	#996433 Archaea
tac	range	#996433 Archaea
afu	range	#996433 Archaea
hal	range	#996433 Archaea
mac	range	#996433 Archaea
mma	range	#996433 Archaea
pfu	range	#996433 Archaea
pho	range	#996433 Archaea
pab	range	#996433 Archaea
mth	range	#996433 Archaea
mka	range	#996433 Archaea
mmp	range	#996433 Archaea
mja	range	#996433 Archaea
.....
```

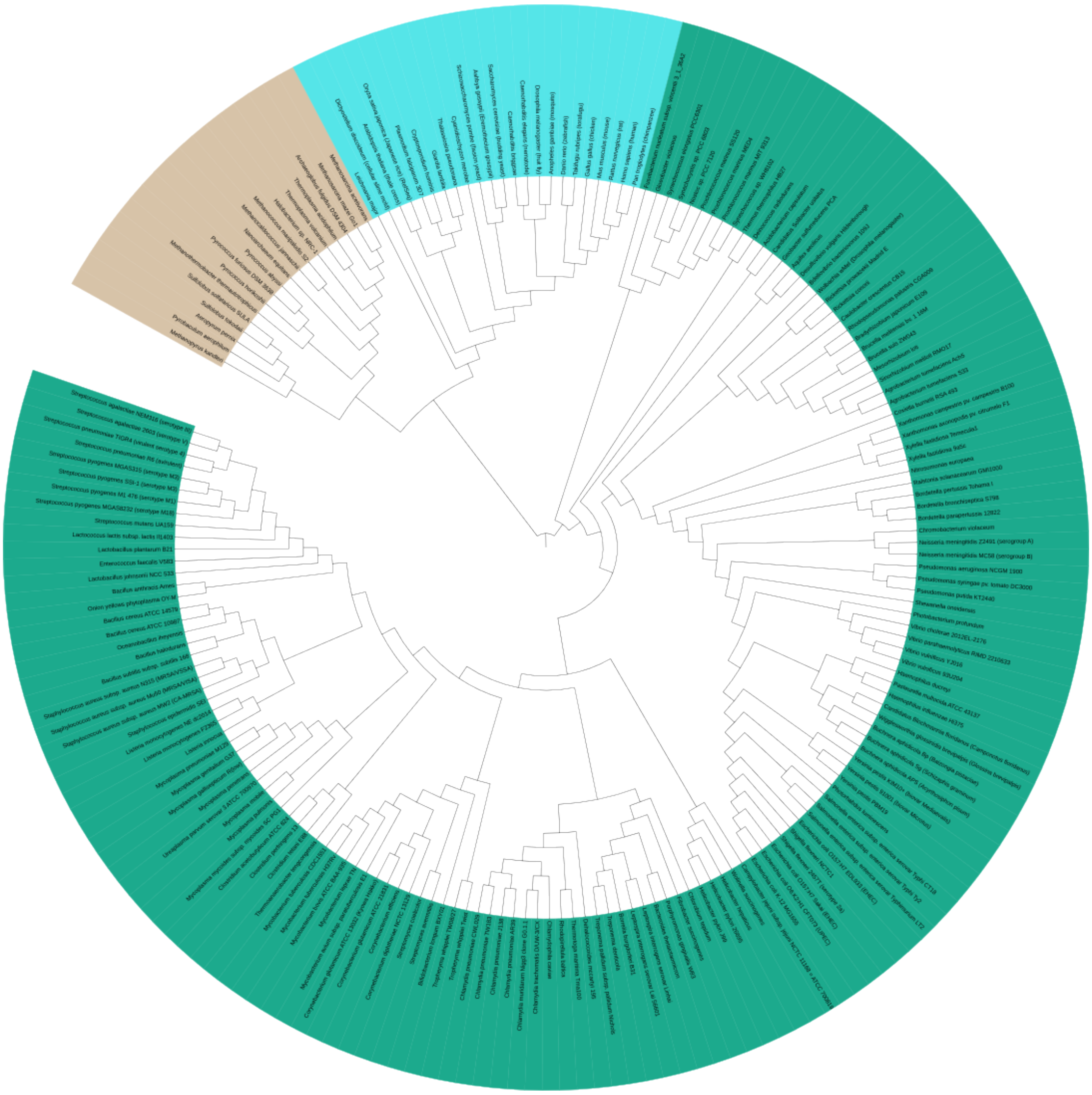

Reconstruct the tree-of-life by the SSU rRNA method

Concept

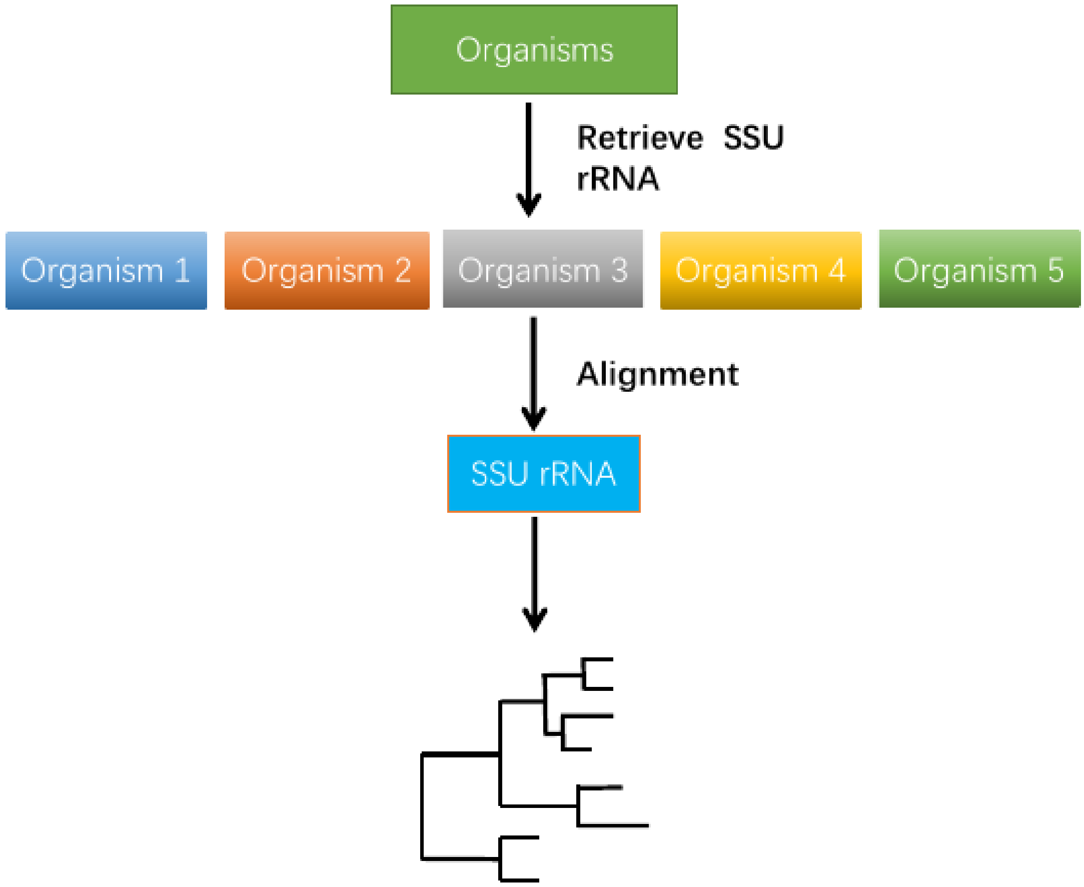

PhySpeTree provides `--srna` option to reconstruct species trees by the SSU rRNA method. The concept of this method is to align SSU rRNA sequences and then reconstruct the specie tree.

1. Prepare input species

```
$ wget "https://yangfangs.github.io/physpetools/example/191speciesnames.txt"

--2016-10-30 15:26:06--  https://yangfangs.github.io/physpetools/example/191s
peciesnames.txt
Resolving yangfangs.github.io (yangfangs.github.io)... 151.101.48.133
Connecting to yangfangs.github.io (yangfangs.github.io)|151.101.48.133|:44
3... connected.
HTTP request sent, awaiting response... 200 OK
Length: 773 [text/plain]
```

```
Saving to: ‘191speciesnames.txt’


$ cat 191speciesnames.txt
neq
pai
ape
sto
ssoa
tvo
tac
afu
.....
```

2. Reconstruct the species tree

```
$ PhySpeTree autobuild -i 191speciesnames.txt -o 191_rna --srna
Loading organisms names success.....


The result are store in:191_rna


Now loading data and constructing phylogenetic tree......
2016-10-30 15:40:17,107 Checking organisms INFO: The organism: neq can't match
 in SSU rRNA database
2016-10-30 15:40:17,107 Checking organisms INFO: The organism: ape can't match
 in SSU rRNA database
2016-10-30 15:40:17,107 Checking organisms INFO: The organism: tac can't match
 in SSU rRNA database
2016-10-30 15:40:17,107 Checking organisms INFO: The organism: mmp can't match
 in SSU rRNA database
2016-10-30 15:40:17,107 Checking organisms INFO: The organism: gla can't match
 in SSU rRNA database
2016-10-30 15:40:17,107 Checking organisms INFO: The organism: tps can't match
 in SSU rRNA database
```

```
2016-10-30 15:40:17,107 Checking organisms INFO: The organism: cho can't match
 in SSU rRNA database

2016-10-30 15:40:17,107 Checking organisms INFO: The organism: ddi can't match
 in SSU rRNA database

2016-10-30 15:40:17,107 Checking organisms INFO: The organism: spo can't match
 in SSU rRNA database

2016-10-30 15:40:17,107 Checking organisms INFO: The organism: aga can't match
 in SSU rRNA database

2016-10-30 15:40:17,107 Checking organisms INFO: The organism: tru can't match
 in SSU rRNA database

2016-10-30 15:40:17,107 Checking organisms INFO: The organism: mpu can't match
 in SSU rRNA database

2016-10-30 15:40:17,107 Checking organisms INFO: The organism: lin can't match
 in SSU rRNA database

2016-10-30 15:40:17,107 Checking organisms INFO: The organism: ban can't match
 in SSU rRNA database

2016-10-30 15:40:17,107 Checking organisms INFO: The organism: bce can't match
 in SSU rRNA database

2016-10-30 15:40:17,108 Checking organisms INFO: The organism: ljo can't match
 in SSU rRNA database

2016-10-30 15:40:17,108 Checking organisms INFO: The organism: san can't match
 in SSU rRNA database

2016-10-30 15:40:17,108 Checking organisms INFO: The organism: spg can't match
 in SSU rRNA database

2016-10-30 15:40:17,108 Checking organisms INFO: The organism: ges can't match
 in SSU rRNA database

2016-10-30 15:40:17,108 Checking organisms INFO: The organism: lis can't match
 in SSU rRNA database

2016-10-30 15:40:17,108 Checking organisms INFO: The organism: sco can't match
 in SSU rRNA database

2016-10-30 15:40:17,108 Checking organisms INFO: The organism: cdi can't match
 in SSU rRNA database

2016-10-30 15:40:17,108 Checking organisms INFO: The organism: mle can't match
 in SSU rRNA database

2016-10-30 15:40:17,108 Checking organisms INFO: The organism: wsu can't match
 in SSU rRNA database
```

```
2016-10-30 15:40:17,108 Checking organisms INFO: The organism: rpr can't match
 in SSU rRNA database

2016-10-30 15:40:17,108 Checking organisms INFO: The organism: bpe can't match
 in SSU rRNA database

2016-10-30 15:40:17,108 Checking organisms INFO: The organism: bpa can't match
 in SSU rRNA database

2016-10-30 15:40:17,108 Checking organisms INFO: The organism: ppr can't match
 in SSU rRNA database

2016-10-30 15:40:17,108 Checking organisms WARNING: These species can't match
in SSU rRNA database so removing and reconstructing phylogenetic tree.

2016-10-30 15:40:17,108 SSU rRNA DB INFO: Read organisms names success

2016-10-30 15:40:24,505 SSU rRNA DB INFO: Retrieve and download of organism 'p
ai' SSU rRNA sequence was successful

2016-10-30 15:40:26,960 SSU rRNA DB INFO: Retrieve and download of organism 's
to' SSU rRNA sequence was successful

2016-10-30 15:40:29,313 SSU rRNA DB INFO: Retrieve and download of organism 's
soa' SSU rRNA sequence was successful

2016-10-30 15:40:32,387 SSU rRNA DB INFO: Retrieve and download of organism 't
vo' SSU rRNA sequence was successful

2016-10-30 15:40:35,158 SSU rRNA DB INFO: Retrieve and download of organism 'a
fu' SSU rRNA sequence was successful

2016-10-30 15:40:37,507 SSU rRNA DB INFO: Retrieve and download of organism 'h
al' SSU rRNA sequence was successful

2016-10-30 15:40:41,282 SSU rRNA DB INFO: Retrieve and download of organism 'm
ac' SSU rRNA sequence was successful

......
```

3. Get the output tree

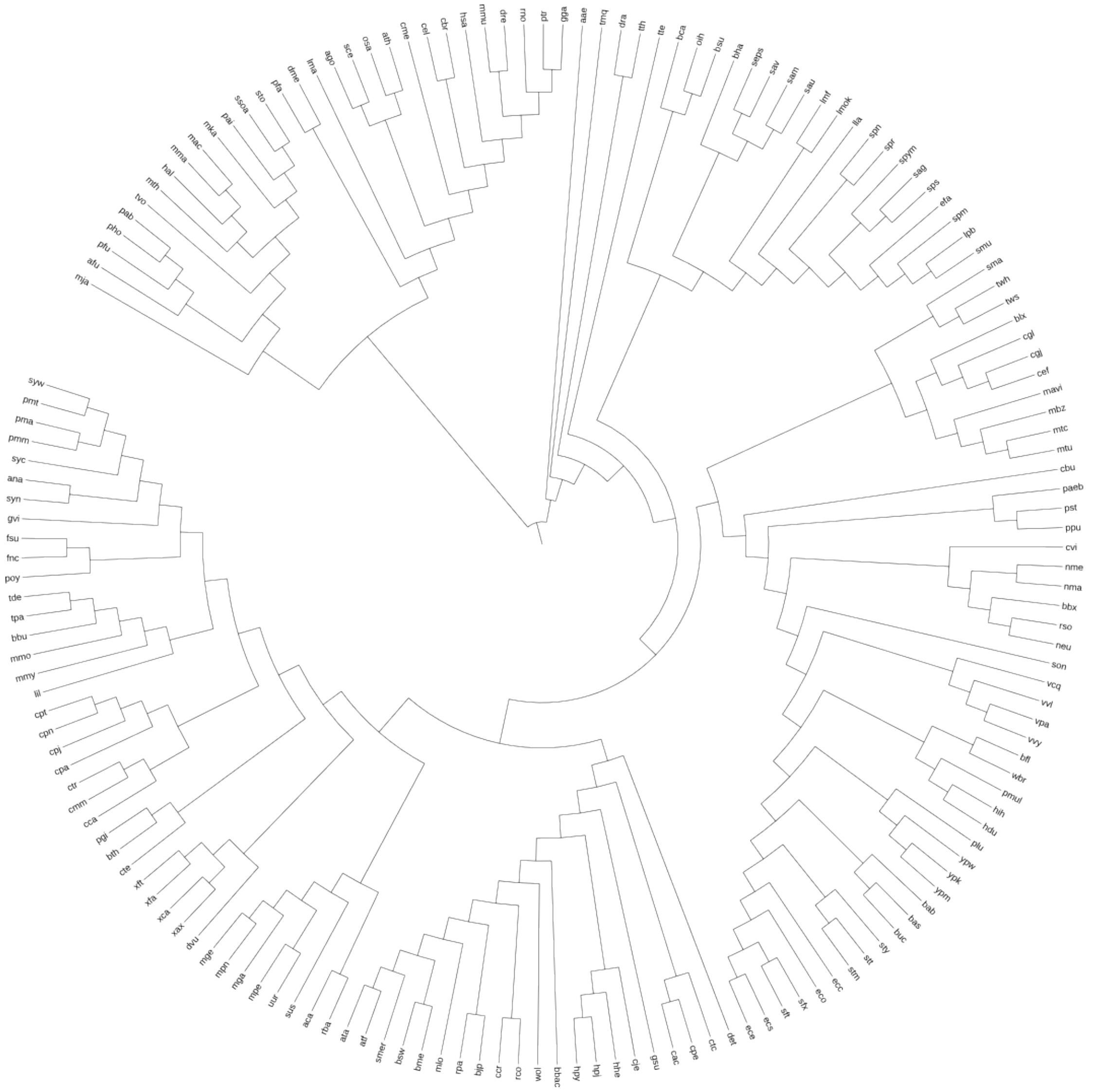

4. Annotate and view the tree

Use the `--labels` option to change abbreviated species names to full names:

```
$ PhySpeTree iview -i 191speciesnames.txt --labels

Change abbreviation names to full names complete

change labels file was save in iview/labels.txt



$ cd iview



$ cat labels.txt
```

```
LABELS

SEPARATOR TAB

DATA

neq	Nanoarchaeum equitans

pai	Pyrobaculum aerophilum

ape	Aeropyrum pernix

sto	Sulfolobus tokodaii

ssoa	Sulfolobus solfataricus SULA

tvo	Thermoplasma volcanium

tac	Thermoplasma acidophilum

afu	Archaeoglobus fulgidus DSM 4304

hal	Halobacterium sp. NRC-1

mac	Methanosarcina acetivorans

mma	Methanosarcina mazei Go1

pfu	Pyrococcus furiosus DSM 3638

pho	Pyrococcus horikoshii

pab	Pyrococcus abyssi

mth	Methanothermobacter thermautotrophicus

mka	Methanopyrus kandleri

mmp	Methanococcus maripaludis S2

.....
```

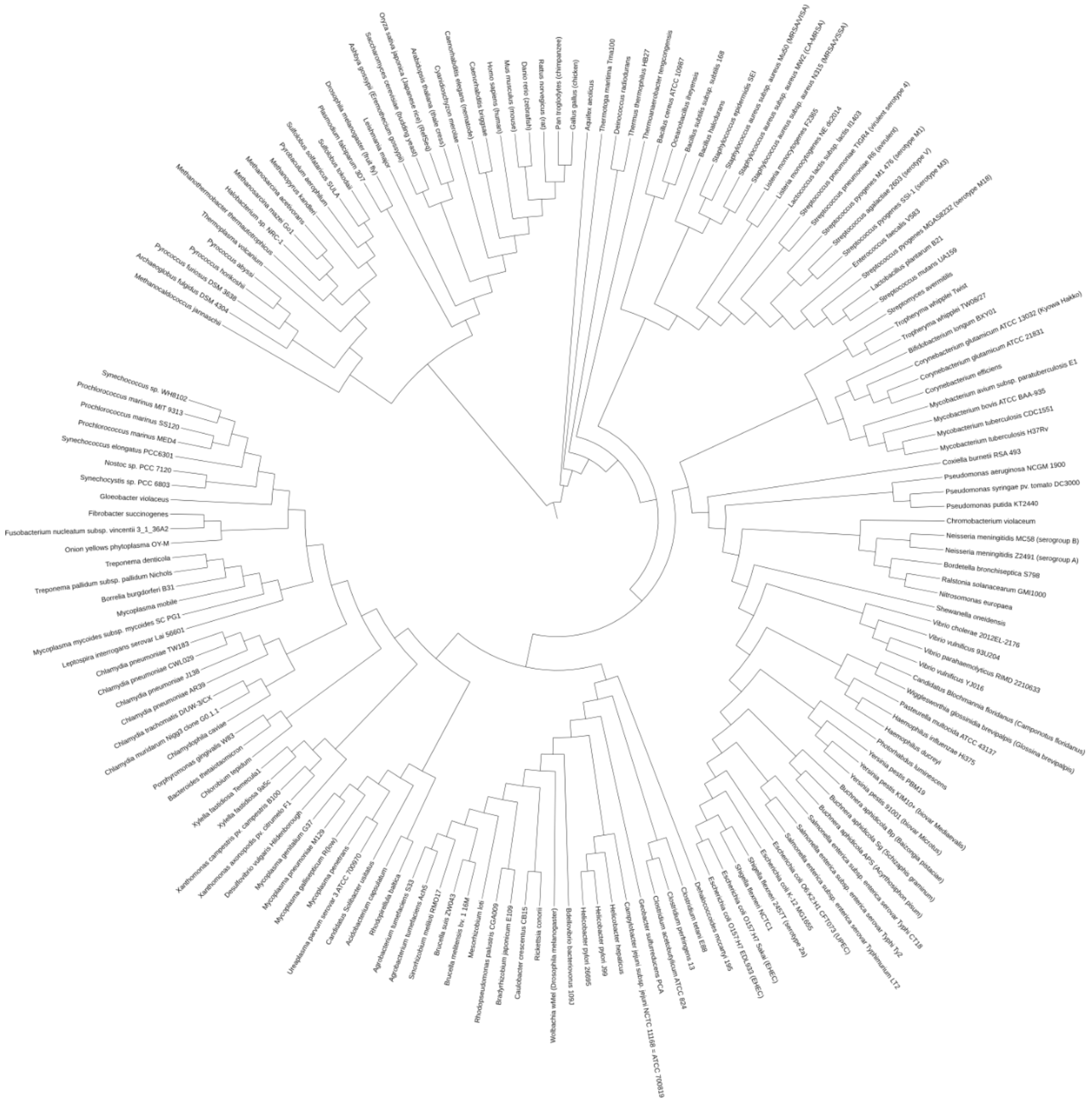

Color branch ranges by phylum:

```
$ PhySpeTree iview -i 191speciesnames.txt -o iview --range -a phylum

Color range by phylum was complete.

Color range annotation was save in iview/range_color_by_phylum.txt



$ cd iview



$ cat range_color_by_phylum.txt

TREE_COLORS

SEPARATOR TAB

DATA
```

```
neq     range   #996433 Archaea

pai     range   #996433 Archaea

ape     range   #996433 Archaea

sto     range   #996433 Archaea

ssoa    range   #996433 Archaea

tvo     range   #996433 Archaea

tac     range   #996433 Archaea

afu     range   #996433 Archaea

hal     range   #996433 Archaea

mac     range   #996433 Archaea

mma     range   #996433 Archaea

pfu     range   #996433 Archaea

pho     range   #996433 Archaea

pab     range   #996433 Archaea

mth     range   #996433 Archaea

mka     range   #996433 Archaea

mmp     range   #996433 Archaea

mja     range   #996433 Archaea

.....
```

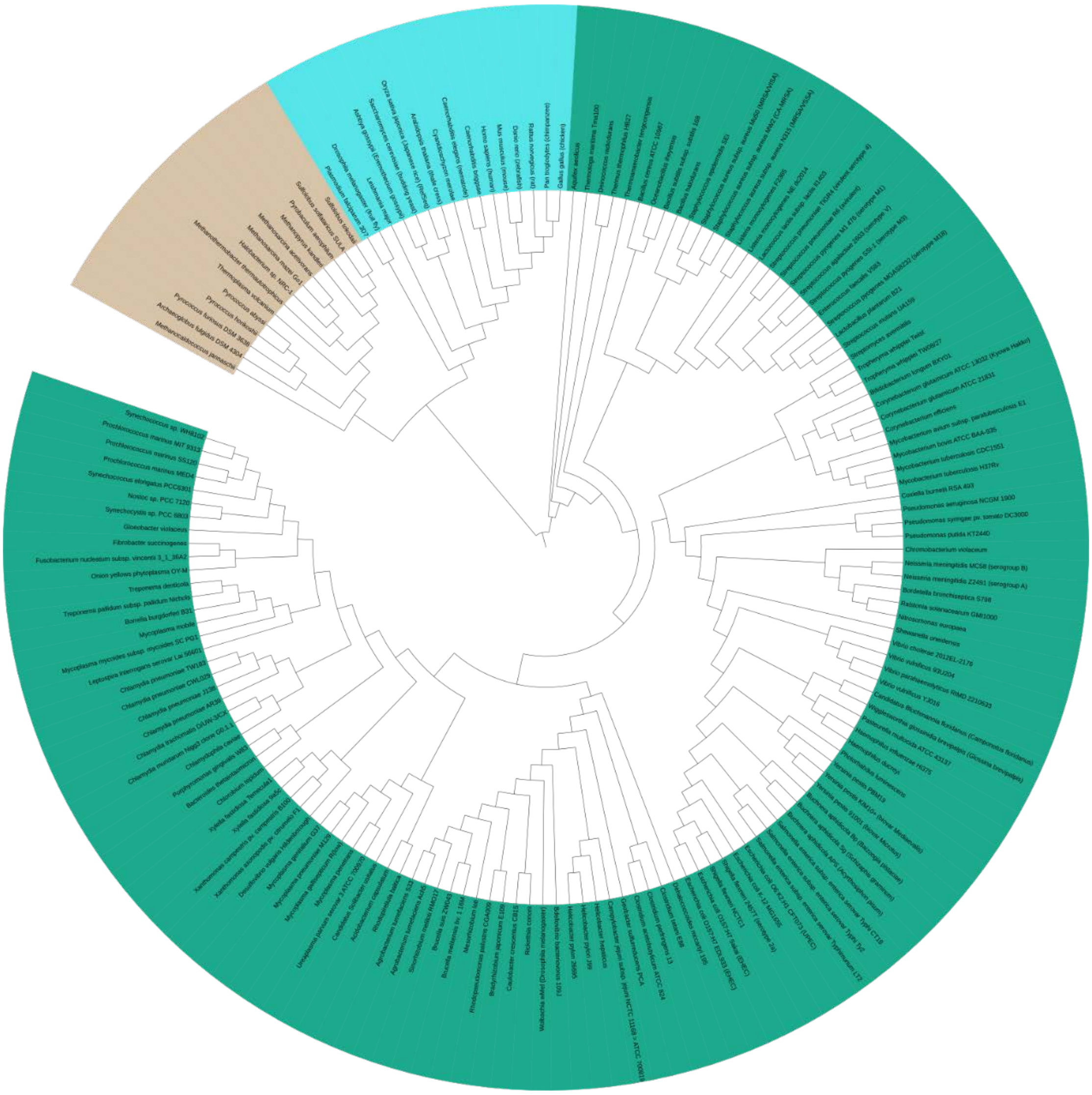

Color branch ranges by class:

```
$ PhySpeTree iview -i 191speciesnames.txt --range -a class

Color range by class was complete.

Color range annotation was save in iview/range_color_by_class.txt



$ cd iview



$ cat range_color_by_class.txt

TREE_COLORS

SEPARATOR TAB

DATA
```

```
neq     range   #4A959E Nanoarchaeota

pai     range   #58CD80 Crenarchaeota

ape     range   #58CD80 Crenarchaeota

sto     range   #58CD80 Crenarchaeota

ssoa    range   #58CD80 Crenarchaeota

tvo     range   #639BB0 Euryarchaeota

tac     range   #639BB0 Euryarchaeota

afu     range   #639BB0 Euryarchaeota

hal     range   #639BB0 Euryarchaeota

mac     range   #639BB0 Euryarchaeota

mma     range   #639BB0 Euryarchaeota

pfu     range   #639BB0 Euryarchaeota

pho     range   #639BB0 Euryarchaeota

pab     range   #639BB0 Euryarchaeota

mth     range   #639BB0 Euryarchaeota

mka     range   #639BB0 Euryarchaeota

mmp     range   #639BB0 Euryarchaeota

mja     range   #639BB0 Euryarchaeota

gla     range   #C5D49E Diplomonads

lma     range   #899DDB Euglenozoa

tps     range   #7DD2ED Stramenopiles

cho     range   #99A01A Alveolates

.....
```

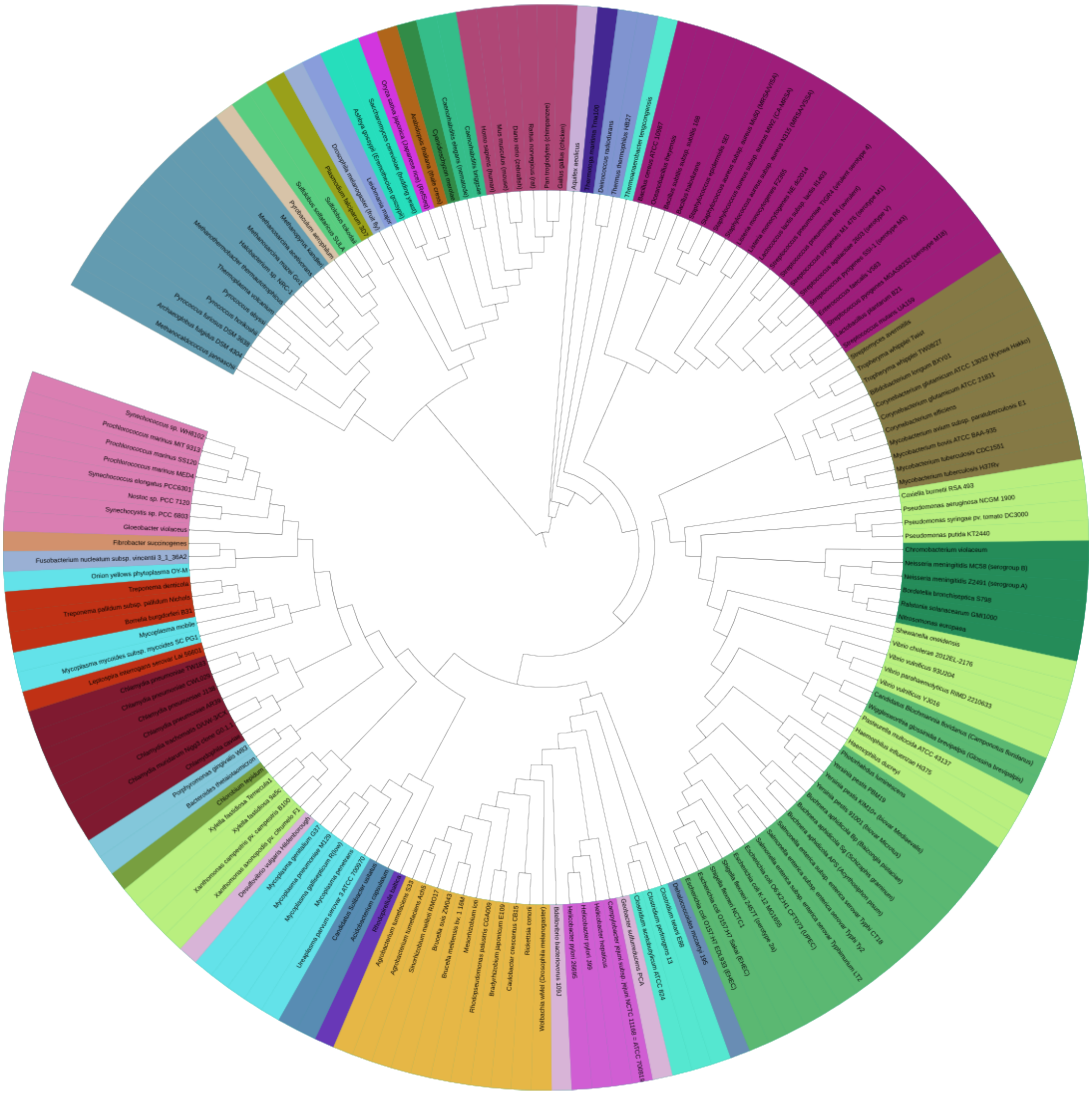

Automatically reconstruct *Plantae* species tree

Here we use PhySpeTree to automatically reconstructed 52 plants species tree by the HCP method.

1. Prepare input species

```
$ wget "https://yangfangs.github.io/physpetools/example/52plantsnames.txt"

--2016-10-30 15:44:25--  https://yangfangs.github.io/physpetools/example/52plantsnames.txt
```

```
Resolving yangfangs.github.io (yangfangs.github.io)... 151.101.48.133

Connecting to yangfangs.github.io (yangfangs.github.io)|151.101.48.133|:44
3... connected.

HTTP request sent, awaiting response... 200 OK

Length: 211 [text/plain]

Saving to: ‘52plantsnames.txt’



52plantsnames.txt          100%[=============================================
==>]     211  --.-KB/s    in 0s



2016-10-30 15:44:27 (120 MB/s) - ‘52plantsnames.txt’ saved [211/211]



$ cat 52plantsnames.txt

aly

ath

atr

bdi

bpg

brp

bvg

cam

ccp

cic

cit

cme

cmo

.....
```

2. Reconstruct the *Plantae* species tree

```
$ PhySpeTree autobuild -i 52plantsnames.txt -o 52plant_pro --hcp -t 6
```

```
Loading organisms names success.....


The result are store in:52plant_pro


Now loading data and constructing phylogenetic tree......

2016-10-30 15:48:23,420 KEGG INDEX DB INFO: Read organisms names success

2016-10-30 15:48:36,020 KEGG INDEX DB INFO: Retrieve and download of highly co
nserved protein 'Leucyl-tRNA synthetase' was successful store in p1.fasta file

2016-10-30 15:48:42,369 KEGG INDEX DB INFO: Retrieve and download of highly co
nserved protein 'Metal-dependent proteases with chaperone activity' was succes
sful store in p2.fasta file

2016-10-30 15:48:46,774 KEGG INDEX DB INFO: Retrieve and download of highly co
nserved protein 'Phenylalanine-tRNA synthethase alpha subunit' was successful
store in p3.fasta file

2016-10-30 15:48:50,559 KEGG INDEX DB INFO: Retrieve and download of highly co
nserved protein 'Preprotein translocase subunit SecY' was successful store in
p4.fasta file

2016-10-30 15:48:54,153 KEGG INDEX DB INFO: Retrieve and download of highly co
nserved protein 'Ribosomal protein L15' was successful store in p5.fasta file

2016-10-30 15:48:57,631 KEGG INDEX DB INFO: Retrieve and download of highly co
nserved protein 'Ribosomal protein L16/L10E' was successful store in p6.fasta
file

2016-10-30 15:49:01,311 KEGG INDEX DB INFO: Retrieve and download of highly co
nserved protein 'Ribosomal protein L18' was successful store in p7.fasta file

2016-10-30 15:49:09,038 KEGG INDEX DB INFO: Retrieve and download of highly co
nserved protein 'Ribosomal protein L22' was successful store in p8.fasta file

2016-10-30 15:49:18,417 KEGG INDEX DB INFO: Retrieve and download of highly co
nserved protein 'Ribosomal protein L3' was successful store in p9.fasta file

2016-10-30 15:49:24,771 KEGG INDEX DB INFO: Retrieve and download of highly co
nserved protein 'Ribosomal protein L5' was successful store in p10.fasta file

2016-10-30 15:49:31,840 KEGG INDEX DB INFO: Retrieve and download of highly co
nserved protein 'Ribosomal protein L6P/L9E' was successful store in p11.fasta
file

2016-10-30 15:49:36,652 KEGG INDEX DB INFO: Retrieve and download of highly co
nserved protein 'Ribosomal protein S11' was successful store in p12.fasta file
```

```
2016-10-30 15:49:41,464 KEGG INDEX DB INFO: Retrieve and download of highly co
nserved protein 'Ribosomal protein S15P/S13E' was successful store in p13.fast
a file

2016-10-30 15:49:46,272 KEGG INDEX DB INFO: Retrieve and download of highly co
nserved protein 'Ribosomal protein S17' was successful store in p14.fasta file

2016-10-30 15:49:51,186 KEGG INDEX DB INFO: Retrieve and download of highly co
nserved protein 'Ribosomal protein S2' was successful store in p15.fasta file

2016-10-30 15:50:04,278 KEGG INDEX DB INFO: Retrieve and download of highly co
nserved protein 'Ribosomal protein S8' was successful store in p16.fasta file

2016-10-30 15:50:13,914 KEGG INDEX DB INFO: Retrieve and download of highly co
nserved protein 'Seryl-tRNA synthetase' was successful store in p17.fasta file

2016-10-30 15:50:19,753 KEGG INDEX DB INFO: Retrieve and download of highly co
nserved protein 'Ribosomal protein S13' was successful store in p18.fasta file

2016-10-30 15:50:19,753 KEGG INDEX DB INFO: Retrieve from KEGG database 18 hig
hly conserved proteins

......
```

3. Get the output tree

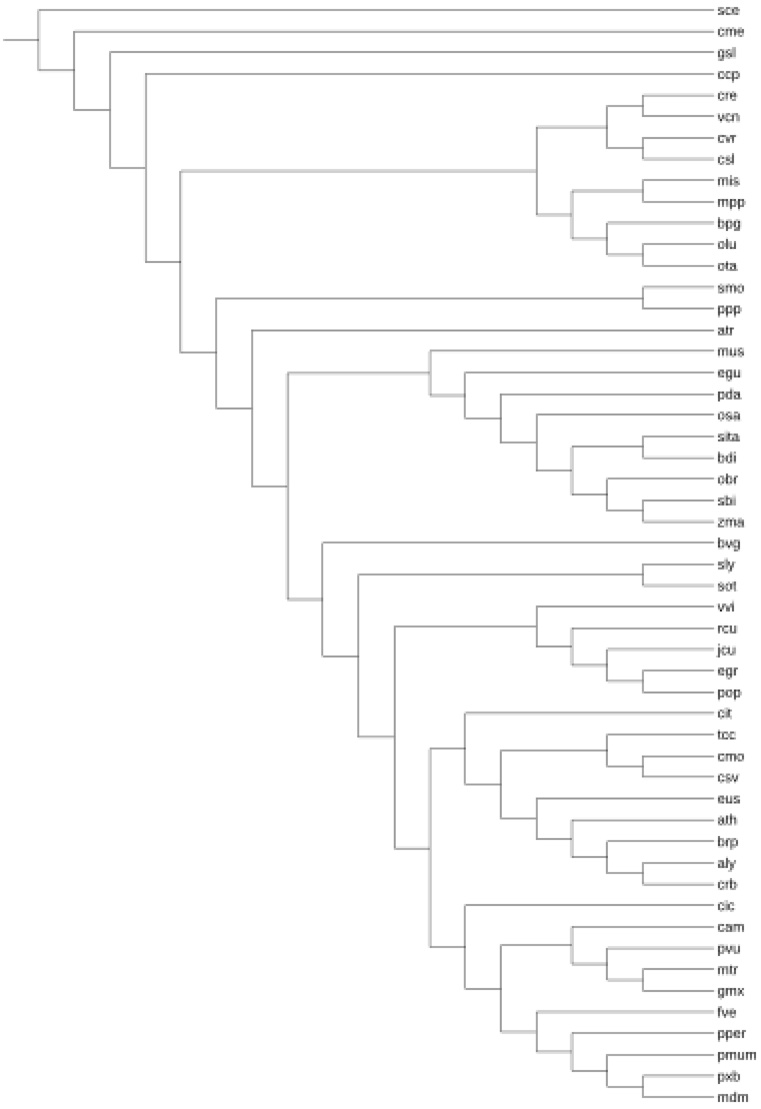

4. Annotate and view the tree

Use the `--labels` option to change abbreviated species names to full names:

```
$ PhySpeTree iview -i 52plantsnames.txt --labels

Change abbreviation names to full names complete

change labels file was save in iview/labels.txt



$ cd iview



$ cat labels.txt
```

```
LABELS

SEPARATOR TAB

DATA

aly	Arabidopsis lyrata (lyrate rockcress)

ath	Arabidopsis thaliana (thale cress)

atr	Amborella trichopoda

bdi	Brachypodium distachyon

bpg	Bathycoccus prasinos

brp	Brassica rapa (field mustard)

bvg	Beta vulgaris (sugar beet)

cam	Cicer arietinum (chickpea)

ccp	Chondrus crispus (carragheen)

cic	Citrus clementina (mandarin orange)

cit	Citrus sinensis (Valencia orange)

cme	Cyanidioschyzon merolae

cmo	Cucumis melo (muskmelon)

crb	Capsella rubella

cre	Chlamydomonas reinhardtii

csl	Coccomyxa subellipsoidea

csv	Cucumis sativus (cucumber)

cvr	Chlorella variabilis

.....
```

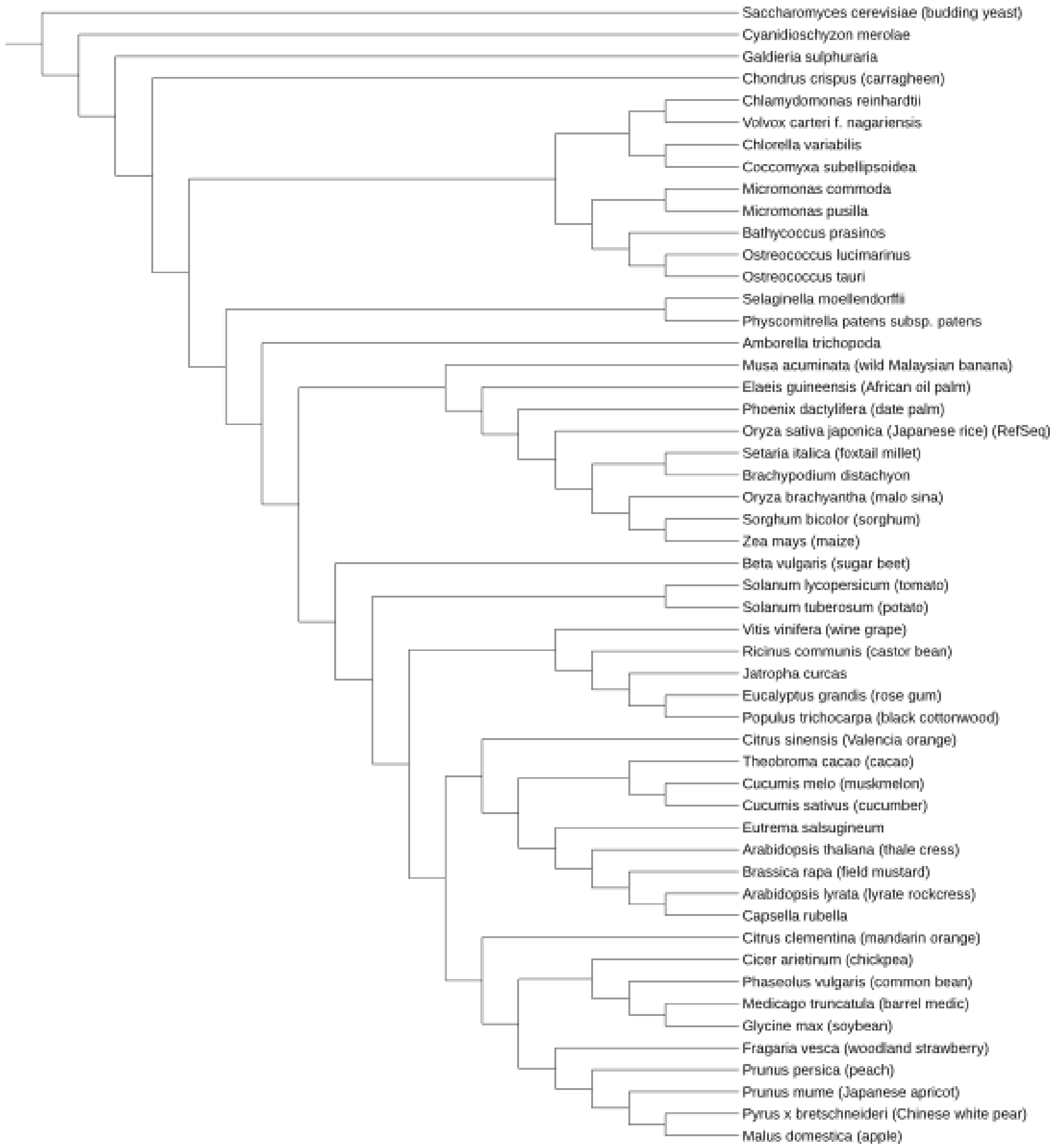

Color branch ranges by class:

```
$ PhySpeTree iview -i 52plantsnames.txt -o iview --range -a phylum

Color range by class was complete.

Color range annotation was save in iview/range_color_by_class.txt

$ cd iview



$ cat range_color_by_class.txt

TREE_COLORS

SEPARATOR TAB

DATA
```

```
aly     range   #1C748D Eudicots

ath     range   #1C748D Eudicots

atr     range   #C4DE94 Basal Magnoliophyta

bdi     range   #854B9F Monocots

bpg     range   #784CE5 Green algae

brp     range   #1C748D Eudicots

bvg     range   #1C748D Eudicots

cam     range   #1C748D Eudicots

ccp     range   #CEDDB3 Red algae

cic     range   #1C748D Eudicots

cit     range   #1C748D Eudicots

cme     range   #CEDDB3 Red algae

cmo     range   #1C748D Eudicots

crb     range   #1C748D Eudicots

cre     range   #784CE5 Green algae

csl     range   #784CE5 Green algae

.....
```

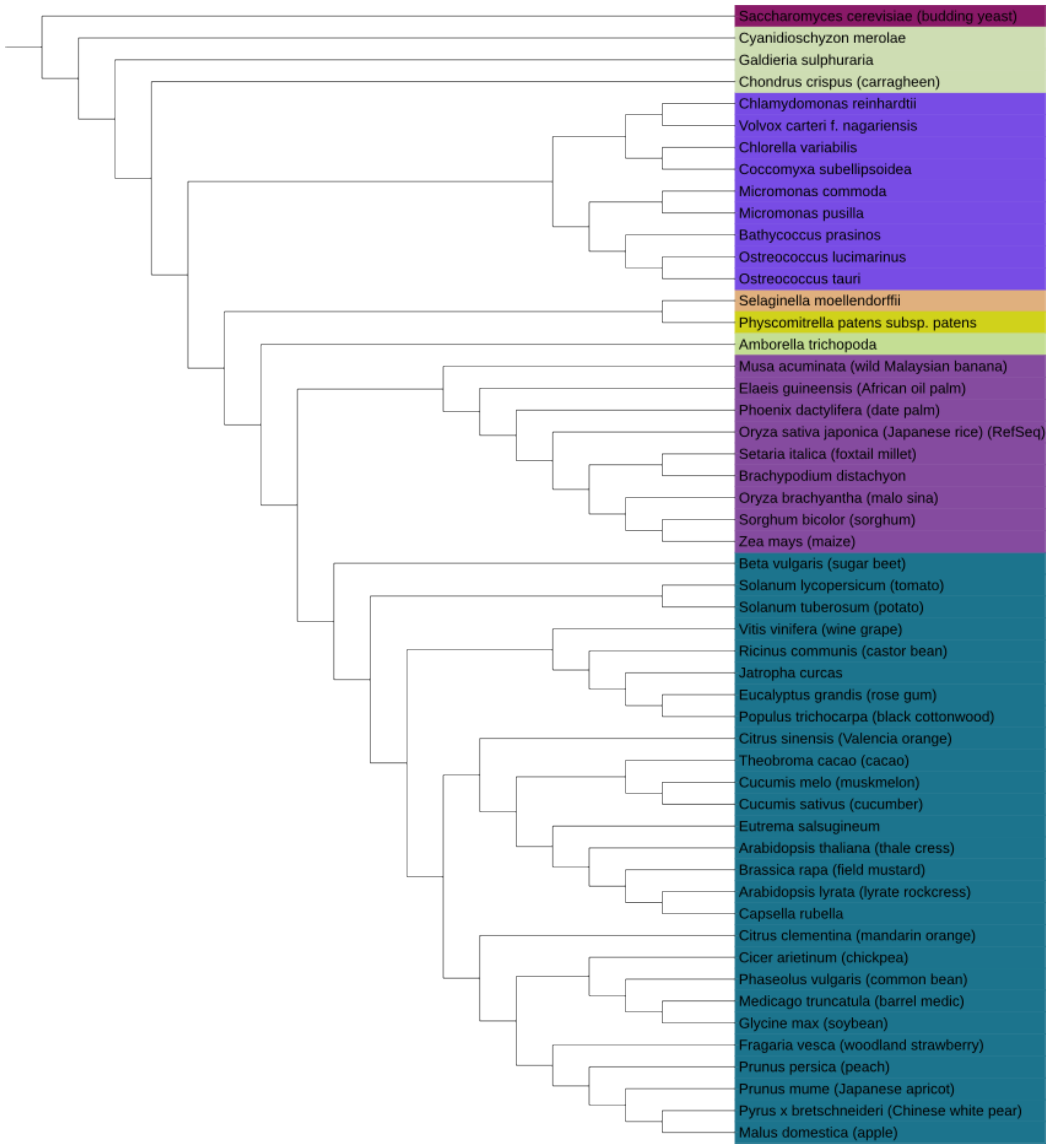

Extend species tree with new organisms

Here we show how to use PhySpeTree to expand insert a a pre-built species tree with new organisms. We insert the newly identified organism *Lokiarchaeum sp. GC14_75* (loki) to the tree-of-life.

Extend species tree by the SSU rRNA method

Concept

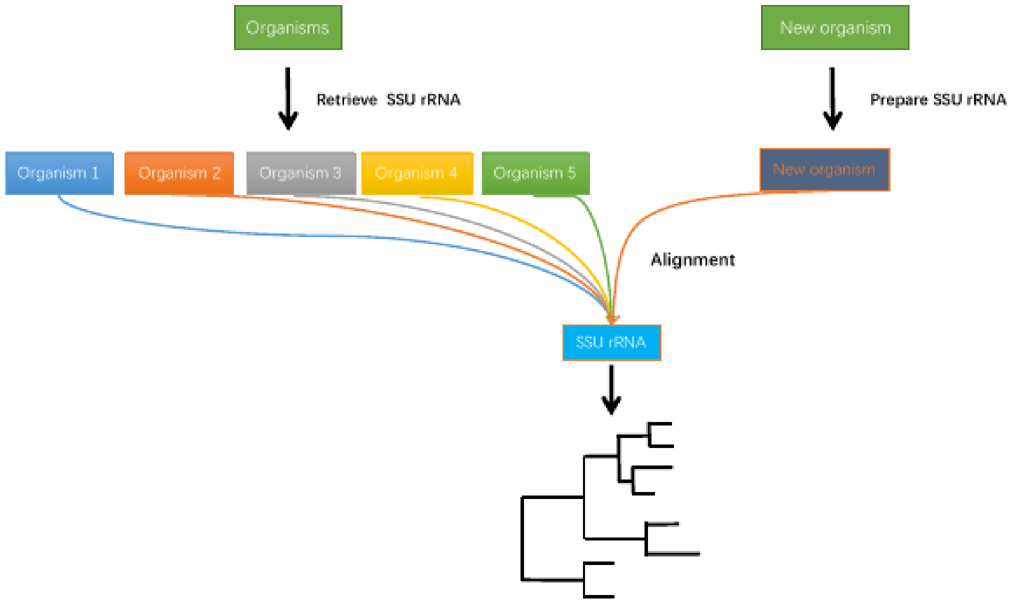

1. Manually prepare the SSU rRNA sequence

We prepare the SSU rRNA sequence of loki and save it in a FASTA format file, for example extend_rna_olki.fasta.

Download the example file:

```
$ wget "https://yangfangs.github.io/physpetools/example/extend_rna_loki.fasta
"


--2016-10-30 15:54:38--  https://yangfangs.github.io/physpetools/example/exte
nd_rna_loki.fasta

Resolving yangfangs.github.io (yangfangs.github.io)... 151.101.24.133

Connecting to yangfangs.github.io (yangfangs.github.io)|151.101.24.133|:44
3... connected.

HTTP request sent, awaiting response... 200 OK

Length: 1200 (1.2K) [application/octet-stream]

Saving to: ‘extend_rna_loki.fasta’


extend_rna_loki.fasta       100%[===========================================
==>]   1.17K  --.-KB/s    in 0s
```

```
2016-10-30 15:54:39 (553 MB/s) - ‘extend_rna_loki.fasta’ saved [1200/1200]
```

check:

```
$ cat extend_rna_olki.fasta

>loki

GAGAUGGGUACUGAGACAACGACCCAGGCCUUACGAGGCGCAGCAGGCGCGAAACCUCCGCAAUACACGAAAGUGU
GACG

GGGUUACCCAAAGUGUUCAAUUAUGAACUGUGGUAGGUGAGUAAUGUUCCCUACUAGAAAGGAGAGGGCAAGGCUG
GUGC

CAGCCGCCGCGGUAAAACCAGCUCUUCAAGUGGUCGGGAUAAUUAUUGGGCUUAAAGUGUCCGUAGCCGGUUUAGU
AAGU

UCCUGGUAAAAUCGGGUAGCUUAACUAUCUGUAUGCUAGGAAUACUGCUAUACUAGAGGACGGGAGAGGUCUGAGG
UACU

ACAGGGGUAGGGGUGAAAUCUUAUAAUCCUUGUAGGACCACCAGUGGCGAAGGCGUCAGACUGGAACGUGCCUGAC
GGUG

AGGGACGAAAGCCAGGGGAGCGAACCGGAUUAGAUACCCGGGUAGUCCUGGCCGUAAACGAUGCAUACUAGGUGAU
GGCA

UGGCCAUGAGCCAUGUCAGUGCCGUAGGGAAACCGUUAAGUGUGCCGCCUGGGAAGUACGGUCGCAAGGCUAAAAC
UUAA

AGGAAUUGGCGGGGGAGCACCACAAGGGGUGAAGCCUGCGGUUCAAUUGGACUCAACGCCGGGAAACUUACCAGGG
GAGA

CAGCAGAAUGAUGGUCAGGUUGACGACCUUACCUGACAAGCUGAGAGGAGGUGCAUGGCCGUCGCCAGUUCGUGCU
GUGA

GGUAUCCUGUUAAGUCAGGCAACGAACGAGAUCCGCACCUUUAUUUGCCAGCAAGAAGUCACGACUUCGUUGGGAA
CACU

AAAGGGACCGCCGUCGAUAAGACGGAGGAAGGAGCGGGCAAAGGCAGGUCAGUAUGCCCCGAAACCCCUGGGCUAC
ACGC

GGGCUGCAAUGGUAUGAACAAUGGGCUGUAACUCCGAAAGGAGAAACCAAUCCCGAAAUCAUAUCUCAGUGGGAAU
UGUC

GGCUGUAACCCGCCGACAUGAACGUGGAAUCCCUAGUAAUCGUGUGUCAUCAUCGCACGGUGAAUACGUCUCUGCU
CCUU

GCACACACCGCCCGUCGCUCCAUCCGAGUGUGCUAAAAAUGAGGUAUGGUCAGUCUGGUCGUAUCGAAUUUCUAGU
AUGC

GAGGGGGGAGAAGUCGUAACAAGGUAGCCGUAGGGGAACCUGCGGCUGGAUCACCUCCU
```

2. Insert loki to the tree-of-life

Update the tree-of-life by combining the `--esrna` and `-e` options.

```
$ PhySpeTree autobuild -i 191speciesnames.txt -o extend_rna -e extend_rna_lok
i.fasta --esrna -t 6


Loading organisms names success.....


The result are store in:extend_rna


Now loading data and constructing phylogenetic tree......

2016-10-30 16:10:01,551 Checking organisms INFO: The organism: neq can't match
 in SSU rRNA database

2016-10-30 16:10:01,551 Checking organisms INFO: The organism: ape can't match
 in SSU rRNA database

2016-10-30 16:10:01,551 Checking organisms INFO: The organism: tac can't match
 in SSU rRNA database

2016-10-30 16:10:01,551 Checking organisms INFO: The organism: mmp can't match
 in SSU rRNA database

2016-10-30 16:10:01,551 Checking organisms INFO: The organism: gla can't match
 in SSU rRNA database

2016-10-30 16:10:01,551 Checking organisms INFO: The organism: tps can't match
 in SSU rRNA database

2016-10-30 16:10:01,551 Checking organisms INFO: The organism: cho can't match
 in SSU rRNA database

2016-10-30 16:10:01,551 Checking organisms INFO: The organism: ddi can't match
 in SSU rRNA database

2016-10-30 16:10:01,551 Checking organisms INFO: The organism: spo can't match
 in SSU rRNA database

2016-10-30 16:10:01,552 Checking organisms INFO: The organism: aga can't match
 in SSU rRNA database

2016-10-30 16:10:01,552 Checking organisms INFO: The organism: tru can't match
 in SSU rRNA database
```

```
2016-10-30 16:10:01,552 Checking organisms INFO: The organism: mpu can't match
 in SSU rRNA database

2016-10-30 16:10:01,552 Checking organisms INFO: The organism: lin can't match
 in SSU rRNA database

2016-10-30 16:10:01,552 Checking organisms INFO: The organism: ban can't match
 in SSU rRNA database

2016-10-30 16:10:01,552 Checking organisms INFO: The organism: bce can't match
 in SSU rRNA database

2016-10-30 16:10:01,552 Checking organisms INFO: The organism: ljo can't match
 in SSU rRNA database

2016-10-30 16:10:01,552 Checking organisms INFO: The organism: san can't match
 in SSU rRNA database

2016-10-30 16:10:01,552 Checking organisms INFO: The organism: spg can't match
 in SSU rRNA database

2016-10-30 16:10:01,552 Checking organisms INFO: The organism: ges can't match
 in SSU rRNA database

2016-10-30 16:10:01,552 Checking organisms INFO: The organism: lis can't match
 in SSU rRNA database

2016-10-30 16:10:01,552 Checking organisms INFO: The organism: sco can't match
 in SSU rRNA database

2016-10-30 16:10:01,552 Checking organisms INFO: The organism: cdi can't match
 in SSU rRNA database

2016-10-30 16:10:01,552 Checking organisms INFO: The organism: mle can't match
 in SSU rRNA database

2016-10-30 16:10:01,552 Checking organisms INFO: The organism: wsu can't match
 in SSU rRNA database

2016-10-30 16:10:01,552 Checking organisms INFO: The organism: rpr can't match
 in SSU rRNA database

2016-10-30 16:10:01,552 Checking organisms INFO: The organism: bpe can't match
 in SSU rRNA database

2016-10-30 16:10:01,552 Checking organisms INFO: The organism: bpa can't match
 in SSU rRNA database

2016-10-30 16:10:01,552 Checking organisms INFO: The organism: ppr can't match
 in SSU rRNA database

2016-10-30 16:10:01,552 Checking organisms WARNING: These species can't match
in SSU rRNA database so removing and reconstructing phylogenetic tree.
```

```
2016-10-30 16:10:01,552 SSU rRNA DB INFO: Read organisms names success

2016-10-30 16:10:02,263 SSU rRNA DB INFO: Retrieve and download of organism 'pai' SSU rRNA sequence was successful

2016-10-30 16:10:02,468 SSU rRNA DB INFO: Retrieve and download of organism 'sto' SSU rRNA sequence was successful

2016-10-30 16:10:02,672 SSU rRNA DB INFO: Retrieve and download of organism 'ssoa' SSU rRNA sequence was successful

2016-10-30 16:10:02,878 SSU rRNA DB INFO: Retrieve and download of organism 'tvo' SSU rRNA sequence was successful

2016-10-30 16:10:04,408 SSU rRNA DB INFO: Retrieve and download of organism 'afu' SSU rRNA sequence was successful

2016-10-30 16:10:04,615 SSU rRNA DB INFO: Retrieve and download of organism 'hal' SSU rRNA sequence was successful

2016-10-30 16:10:05,085 SSU rRNA DB INFO: Retrieve and download of organism 'mac' SSU rRNA sequence was successful

2016-10-30 16:10:05,638 SSU rRNA DB INFO: Retrieve and download of organism 'mma' SSU rRNA sequence was successful

2016-10-30 16:10:05,841 SSU rRNA DB INFO: Retrieve and download of organism 'pfu' SSU rRNA sequence was successful

2016-10-30 16:10:06,086 SSU rRNA DB INFO: Retrieve and download of organism 'pho' SSU rRNA sequence was successful

2016-10-30 16:10:06,590 SSU rRNA DB INFO: Retrieve and download of organism 'pab' SSU rRNA sequence was successful

2016-10-30 16:10:07,100 SSU rRNA DB INFO: Retrieve and download of organism 'mth' SSU rRNA sequence was successful

2016-10-30 16:10:07,303 SSU rRNA DB INFO: Retrieve and download of organism 'mka' SSU rRNA sequence was successful

.....
```

3. Annotate and view the tree

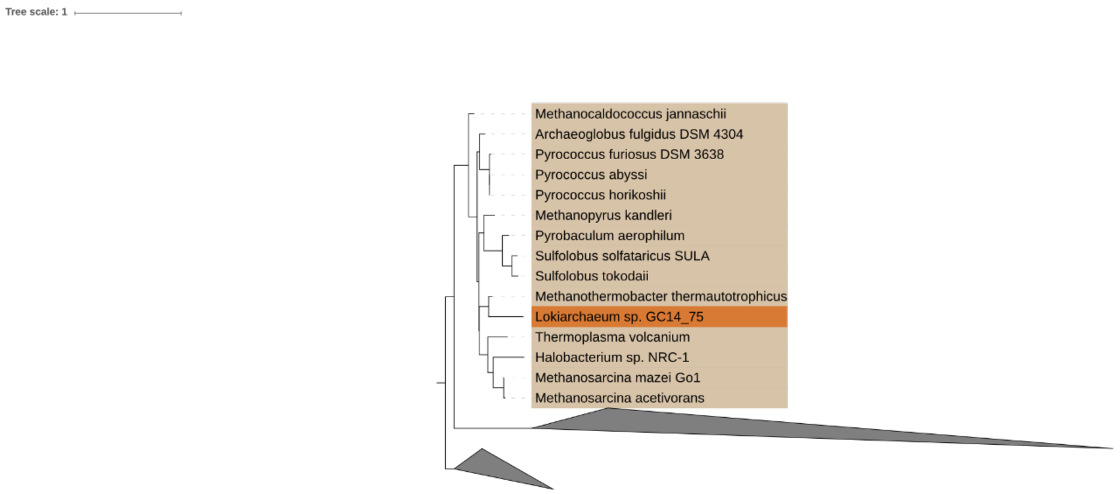

Extend species tree by the HCP method

- **Concept**

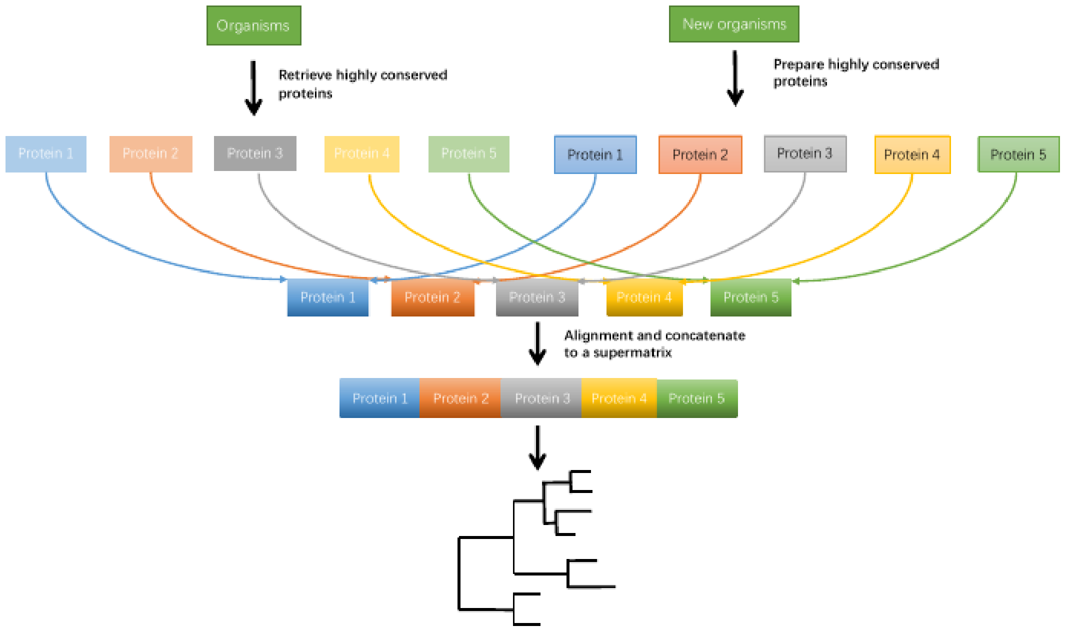

1. Identify classes of HCP

Use the `check` module to identify what classes of HCP should be prepapred.

```
$ PhySpeTree check -i 191speciesnames.txt --ehcp
'Ribosomal protein L1' ----------------------------------> p1.fasta
```

```
'Leucyl-tRNA synthetase' ----------------------------------> p2.fasta

'Ribosomal protein L14' ----------------------------------> p3.fasta

'Ribosomal protein L5' ----------------------------------> p4.fasta

'Ribosomal protein S7' ----------------------------------> p5.fasta

'Arginyl-tRNA synthetase' ----------------------------------> p6.fasta

Checked extend highly conserved proteins is completed.
Checked result was store in check/PhySpeTree_echp_extend.txt
......
```

2. Manually prepare HCP sequences

According to `check` results, we prepare 6 classes of HCP and store the sequences (p1 ~ p6) into FASTA format files, for example highly_conserved_protein_loki

Download the example file:

```
$ wget "https://yangfangs.github.io/physpetools/example/highly_conserved_protein_loki.tar.gz"

--2016-10-30 16:36:48--  https://yangfangs.github.io/physpetools/example/highly_conserved_protein_loki.tar.gz
Resolving yangfangs.github.io (yangfangs.github.io)... 151.101.48.133
Connecting to yangfangs.github.io (yangfangs.github.io)|151.101.48.133|:443... connected.
HTTP request sent, awaiting response... 200 OK
Length: 1810 (1.8K) [application/octet-stream]
Saving to: ‘highly_conserved_protein_loki.tar.gz’
```

```
highly_conserved_protein_lok 100%[==========================================
===>]   1.77K  --.-KB/s    in 0s


2016-10-30 16:36:50 (623 MB/s) - ‘highly_conserved_protein_loki.tar.gz’ saved
 [1810/1810]

$ tar -zxvf highly_conserved_protein_loki.tar.gz


highly_conserved_protein_loki/

highly_conserved_protein_loki/p1.fasta

highly_conserved_protein_loki/p2.fasta

highly_conserved_protein_loki/p3.fasta

highly_conserved_protein_loki/p4.fasta

highly_conserved_protein_loki/p5.fasta

highly_conserved_protein_loki/p6.fasta


$ cd extend_pro_loki

$ ls

p1.fasta  p2.fasta  p3.fasta  p4.fasta  p5.fasta  p6.fast


$ cat p1.fasta

>loki

MKVDDNLLKQSLNAAIDFSVRKKEGFKDRVRKFDETIDLIINIKDVNLNDPKNRIDKEII

LTNEIVEEEKLNICVIASGEILLEAKKAGVETLDRDALIKLNNEEKKHKKKFAKKYEFFI

VEDKMMRDVARYLARFLGPLGKMPKPFPTGYGIISSPGDLRTAVERYKKVIRIQMKKQPI

IFAKIGKKSMEIDRLFDNMKTVIDFIADQMPHKFNNFKSMYLKSSMGKPIKVTEEFLKSL

EV
```

3. Insert loki to the tree-of-life

```
$ PhySpeTree autobuild -i 191speciesnames.txt -o extend_pro_loki -e highly_con
served_protein_loki --ehcp -t 6

Loading organisms names success.....
```

```
The result are store in:extend_pro_loki


Now loading data and constructing phylogenetic tree......

2016-10-30 18:19:53,951 Checking organisms INFO: The species: ges can't match in KEGG protein index database

2016-10-30 18:19:53,951 Checking organisms WARNING: These species can't match in KEGG protein index database so removed and reconstruct phylogenetic tree.

2016-10-30 18:19:53,951 KEGG INDEX DB INFO: Read organisms names success

2016-10-30 18:20:18,870 KEGG INDEX DB INFO: Retrieve and download of highly conserved protein 'Ribosomal protein L1' was successful store in p1.fasta file

2016-10-30 18:20:46,573 KEGG INDEX DB INFO: Retrieve and download of highly conserved protein 'Leucyl-tRNA synthetase' was successful store in p2.fasta file

2016-10-30 18:21:11,401 KEGG INDEX DB INFO: Retrieve and download of highly conserved protein 'Ribosomal protein L14' was successful store in p3.fasta file

2016-10-30 18:21:36,078 KEGG INDEX DB INFO: Retrieve and download of highly conserved protein 'Ribosomal protein L5' was successful store in p4.fasta file

2016-10-30 18:22:00,454 KEGG INDEX DB INFO: Retrieve and download of highly conserved protein 'Ribosomal protein S7' was successful store in p5.fasta file

2016-10-30 18:22:27,895 KEGG INDEX DB INFO: Retrieve and download of highly conserved protein 'Arginyl-tRNA synthetase' was successful store in p6.fasta file

2016-10-30 18:22:27,895 KEGG INDEX DB INFO: Retrieve from KEGG database 6 highly conserved proteins

......
```

4. Annotate and view the tree

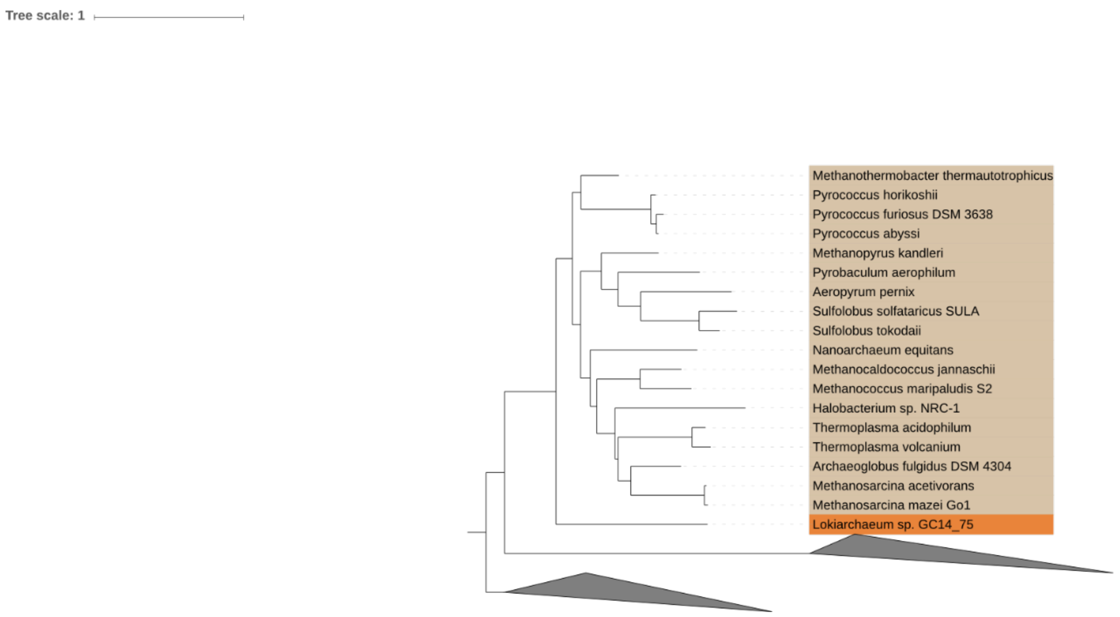

Run PhySpeTree in other operating systems

1. Install the Docker in your operating system

- If you want to use PhySpeTree on other platforms, above all need to install docker on the appropriate platform.
- For Windows OS, you can install Docker for Windows.
- For Mac OS, you can install Docker for Mac.

2. Pull the PhySpeTree image

```
$ docker pull yangfangs/physpetree:v0.3.4

[yangfang@localhost ~]$ docker pull yangfangs/physpetree:v0.3.4

v0.3.4: Pulling from yangfangs/physpetree

Digest: sha256:2fef685db22bd18ae0595884bfd6ba7409b15fbb6b7c579e1817ac007cc46c
fe

Status: Downloaded newer image for yangfangs/physpetree:v0.3.4
```

3. check PhySpeTree image

```
[yangfang@localhost ~]$ docker images
REPOSITORY             TAG                  IMAGE ID            CREATED             SIZE
yangfangs/physpetree   v0.3.4               ebcdaff79f7c        41 hours ago        683MB
```

4. run PhySpeTree image

- You need to run docker with an interactive bash and mount it in a local directory with a working directory in docker (PhySpeTree_work_dir).

```
[yangfang@localhost ~]$ docker run -it -v /home/yangfang/work_dir/:/PhySpeTree_work_dir yangfangs/physpetree:v0.3.4 bash
root@2d68776ac9ad:/PhySpeTree_work_dir#
```

5. Run PhySpeTree in Docker

- Download test file

```
root@2d68776ac9ad:/PhySpeTree_work_dir# wget "https://yangfangs.github.io/physpetools/example/organism_example_list.txt"
--2018-11-21 02:28:28--  https://yangfangs.github.io/physpetools/example/organism_example_list.txt
Resolving yangfangs.github.io (yangfangs.github.io)... 185.199.108.153, 185.199.109.153, 185.199.111.153, ...
Connecting to yangfangs.github.io (yangfangs.github.io)|185.199.108.153|:443... connected.
HTTP request sent, awaiting response... 200 OK
Length: 39 [text/plain]
Saving to: 'organism_example_list.txt'


organism_example_list.txt    100%[=============================================>]      39  --.-KB/s    in 0s
```

```
2018-11-21 02:28:30 (2.00 MB/s) - 'organism_example_list.txt' saved [39/39]
```

- run PhySpeTree

```
root@2d68776ac9ad:/PhySpeTree_work_dir# PhySpeTree autobuild -i organism_example_list.txt --hcp

Loading organism's names success.....



The result are store in:Outdata



Now loading data and constructing phylogenetic tree......

2018-11-21 02:30:29,336 KEGG INDEX DB INFO: Read organisms names success

2018-11-21 02:30:30,652 KEGG INDEX DB INFO: Retrieve and download of highly conserved protein 'Ribosomal protein L1' was successful store in p1.fasta file

2018-11-21 02:30:31,440 KEGG INDEX DB INFO: Retrieve and download of highly conserved protein 'DNA-directed RNA polymerase subunit alpha' was successful store in p2.fasta file

2018-11-21 02:30:32,260 KEGG INDEX DB INFO: Retrieve and download of highly conserved protein 'Leucyl-tRNA synthetase' was successful store in p3.fasta file

2018-11-21 02:30:33,078 KEGG INDEX DB INFO: Retrieve and download of highly conserved protein 'Metal-dependent proteases with chaperone activity' was successful store in p4.fasta file

2018-11-21 02:30:33,896 KEGG INDEX DB INFO: Retrieve and download of highly conserved protein 'Phenylalanine-tRNA synthethase alpha subunit' was successful store in p5.fasta file

2018-11-21 02:30:35,739 KEGG INDEX DB INFO: Retrieve and download of highly conserved protein 'Predicted GTPase probable translation factor' was successful store in p6.fasta file

2018-11-21 02:30:36,222 KEGG INDEX DB INFO: Retrieve and download of highly conserved protein 'Ribosomal protein L11' was successful store in p7.fasta file

2018-11-21 02:30:36,866 KEGG INDEX DB INFO: Retrieve and download of highly conserved protein 'Ribosomal protein L13

.....
```

- All output result are in local directory, named `work_dir`

```
[yangfang@localhost ~]$ cd work_dir/

[yangfang@localhost work_dir]$ ls

log.log  organism_example_list.txt  Outdata  temp
```
